# The modulating role of memory load on language switching in sentence comprehension: evidence from eye movements

**DOI:** 10.1186/s40359-026-04871-1

**Published:** 2026-05-30

**Authors:** Yun Wang, Xinfang Liu, Chang’an Sun

**Affiliations:** 1 School of AI for the Humanities, Zhejiang International Studies University, Hangzhou, China; 2 School of Foreign Languages and Literature, Suzhou University of Science and Technology, Suzhou, China; 3 School of Education, Suzhou University of Science and Technology, Suzhou, China

**Keywords:** Memory load, Sentence comprehension, Language switching, Modulating effect, Eye-tracking

## Abstract

This study employed eye-tracking technology to investigate the effect of memory load on language switching during Chinese-English bilingual sentence comprehension. Using a within-subject experimental design with three levels of load (low, medium, high), two language types (Chinese, English), and two context types (non-switch, switch), the study collected behavioral measures and eye-tracking measures. The results revealed that under high load, switching from English (L2) to Chinese (L1) resulted in the longest reaction times and the highest costs, whereas switching into L2 consistently yielded switch benefits. Eye-tracking data indicated a dissociation between temporal and spatial dimensions at the sentence level, with switch effects demonstrating contrasting patterns. Similar processing patterns were observed at the target word across the early and late stages, with a significant increase in the effect in the latter. These findings suggest that language-switching costs are not fixed but adaptable, with cognitive load modulating the interplay between language and context, highlighting the intricate relationship between domain-general cognitive control and language-specific switching mechanisms.

**Supplementary Information:**

The online version contains supplementary material available at 10.1186/s40359-026-04871-1.

## Highlights

1. High workloads affect L1 and L2 disproportionately.

2. Language switching costs are not fixed, but are adaptable under different loads.

3. A dissociation exists between temporal and spatial dimensions during sentence comprehension.

4. Early and late processing of local target words exhibit a similar pattern, with the latter being more notable.

**Supplementary Information:**

The online version contains supplementary material available at 10.1186/s40359-026-04871-1.

## Introduction

In contemporary globalized contexts, bilingualism and multilingualism are increasingly prevalent. This reality is reflected in two related but distinct phenomena: code-switching and language switching. Code-switching refers to the natural alternation between two or more languages within a single discourse, shaped by sociolinguistic norms and communicative intentions [1]. Language switching, in contrast, is an experimental paradigm that examines the controlled alternation between languages to understand the underlying cognitive control mechanisms [2]. The experimental language-switching paradigm aims to isolate and investigate the cognitive processes involved in code-switching, such as inhibition, monitoring, and task-set reconfiguration. By examining how the cognitive system manages controlled language switches in a laboratory setting, we can gain insights into the processing demands that bilinguals face when naturally alternating between languages in conversation. The present study specifically examines language switching, focusing on how working memory load influences its cognitive demands during real-time sentence comprehension.

A pivotal discovery in language-switching research is the “switch cost,” which indicates decreased accuracy or slower reaction times when switching between languages compared to repeating a language [3]. This cost is a key index of bilingual language control. Early theoretical accounts, such as the Inhibitory Control Model (ICM), posit that this cost primarily reflects the need to inhibit the non-target language lexicon, with the magnitude of inhibition being proportional to a language’s activation level (e.g., the dominant L1 requires stronger inhibition when switching to L2; [4]). Crucially, the switch cost is not fixed; it varies with factors such as contextual predictability [5] and, notably, the direction of the switch (L1-to-L2 vs. L2-to-L1). This asymmetry arises from inherent disparities in language proficiency: L1 processing is typically more automatic, while L2 processing demands more controlled, resource-consuming effort [6]. Importantly, it is worth noting that the classic asymmetric switch cost pattern (i.e., higher costs for the dominant language) has been predominantly observed in language production tasks [7, 8]. In contrast, language comprehension studies have often yielded different patterns, sometimes showing reduced costs, symmetrical costs, or even switch benefits [9, 10]. This production-comprehension distinction suggests that the mechanisms underlying language switching may be task-dependent, requiring further investigation into how cognitive resources, such as working memory, modulate switching during real-time sentence comprehension.

The Adaptive Control Hypothesis (ACH) extends this framework by proposing that bilingual language control is not a static property but a dynamic system that adapts to the interactive context [11]. It suggests that cognitive control processes, including monitoring and inhibition, are regulated in real time in response to task demands and available cognitive resources. Understanding how finite cognitive resources constrain this asymmetric and adaptive control is therefore central to contemporary models of bilingual processing.

A primary candidate for such a resource constraint is working memory load. Working memory, a system characterized by limited capacity for temporary storage and processing [12], plays a crucial role in supporting complex cognition. According to cognitive load theory, consuming resources for one task (e.g., maintaining a memory load) reduces their availability for another [13]. Consequently, high working memory load is expected to hinder the control processes (e.g., inhibition, task-set reconfiguration) that govern efficient language switching [14]. Prior research has shown that cognitive load can exacerbate switching difficulties [15] and affect neural signatures of control [16, 17]. However, a critical gap remains: a comprehensive, real-time analysis of how parametrically varied working memory load interacts with language switching during the dynamic processes of sentence comprehension is still absent. Most studies have focused on isolated words or single-language processing under load [15]. Examining this interaction in a sentential context is vital, as it engages not only lexical-level control but also higher-level semantic integration, processes that may be differentially susceptible to resource depletion and adaptive control mechanisms.

Eye-tracking technology is well-suited to address this gap. It provides a continuous, high-resolution measure of cognitive processing during naturalistic reading [18]. Specific metrics, such as fixation duration (temporal), saccade amplitude (spatial), and pupil size (physiological), are validated indicators of processing effort and cognitive load [19–21]. By analyzing these metrics at both the sentence and target-word levels, we can dissect how memory load modulates switching across different aspects of comprehension.

Therefore, this study employs eye-tracking to investigate the modulating role of working memory load on language-switching during Chinese-English bilingual sentence comprehension. Grounded in the Adaptive Control Hypothesis [11], which emphasizes the context-dependent and resource-adaptive allocation of control, we hypothesize that: (1) Memory load (low, medium, high) will interact with language (L1, L2) and context (non-switch, switch), producing asymmetric switch effects. However, given the production-comprehension distinction, we do not expect the classic production-based asymmetry (higher costs for L1). Instead, we predict that switching into L2 may confer a switch benefit (i.e., faster or more efficient processing) across load levels, reflecting reduced competition from the dominant language in comprehension, whereas switching into L1 may incur a switch cost only when cognitive resources are depleted under high load. (2) The effect of working memory load will manifest differently across processing levels (global sentence integration vs. local lexical access) and stages (early vs. late processing of the target word), as captured by a constellation of eye-tracking measures. Specifically, we predict that high load will impair resource-demanding late-stage semantic integration during switches, and that this impairment will be more pronounced for L2 than for L1.

**Fig. 1:**
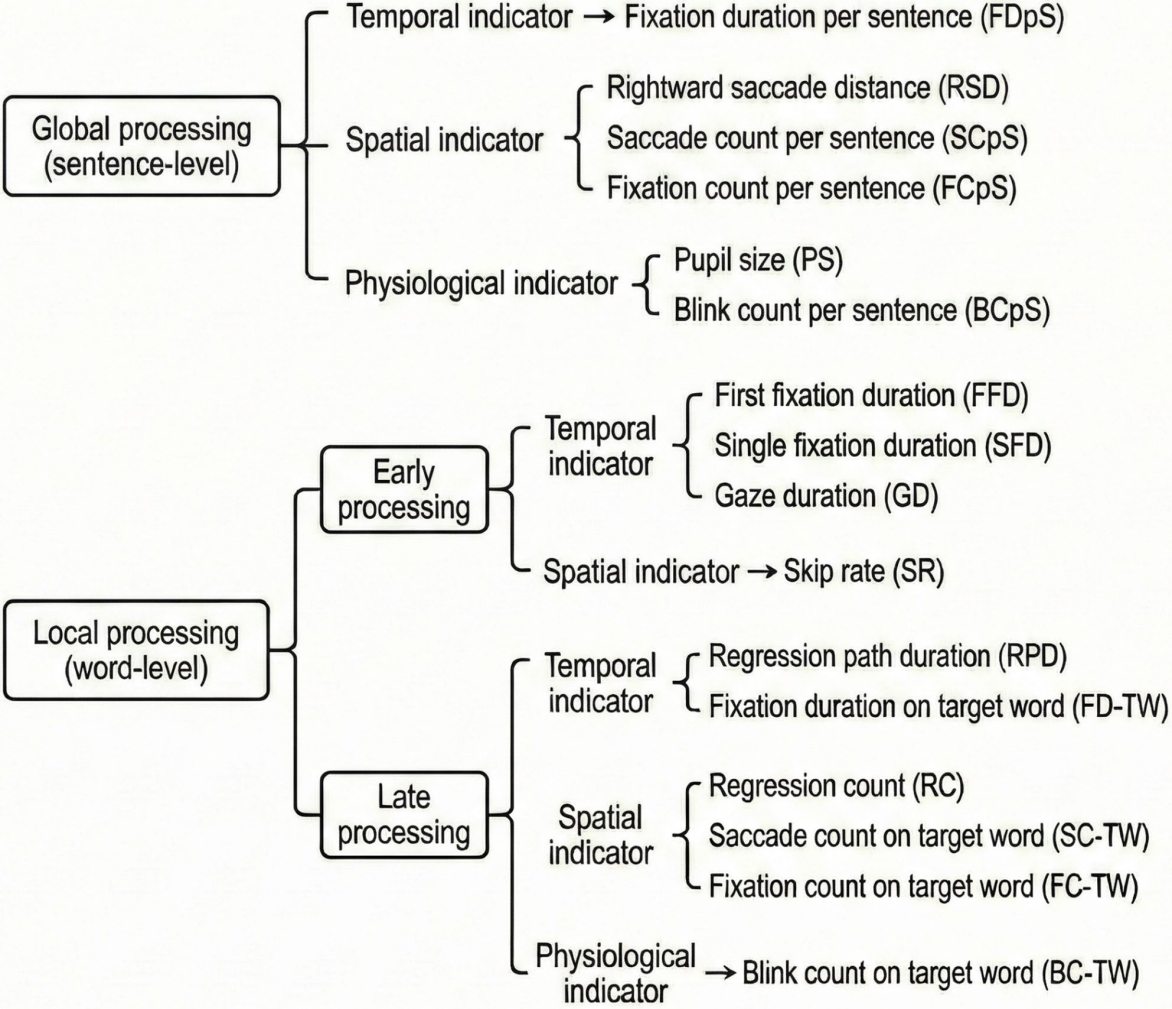
A level- and stage-based framework of eye-tracking metrics

To address these hypotheses, the present study adopts a multilevel research framework grounded in eye-movement research. This framework distinguishes between global (sentence-level) processing, which reflects overall reading fluency and integration, and local (word-level) processing, focused on the target word. Critically, we examine processing across three fundamental dimensions: temporal, spatial, and physiological. The temporal dimension captures the time course of processing, indexed by measures such as fixation durations and regression path durations, which reflect cognitive load, processing speed, and stages of lexical access and semantic integration [22, 23]. The spatial dimension captures the distribution and planning of visual attention, indexed by measures such as saccade amplitudes, fixation counts, and skipping patterns, which reveal readers’ sampling strategies, attentional allocation, and efficiency of information extraction [22, 24]. The physiological dimension reflects autonomic and cognitive arousal, as indexed by pupillary responses and blink rates, providing continuous, implicit markers of cognitive effort and resource mobilization [21, 25]. Local processing is further dissociated into early-stage and late-stage components, indexing initial lexical access and deeper semantic integration/conflict resolution, respectively [22, 26] (see Fig. 1). This hierarchical approach allows for a systematic investigation of whether and how parametrically varied memory load differentially modulates switching effects across distinct levels of representation and stages of processing, thereby testing hypotheses derived from adaptive models of bilingual control.

## Methods

### Participants

Using the More Power 6.0.4 software [27], a sample size of 38 was calculated for a 3 × 2 × 2 within-subject design, with a statistical power of 0.8, an effect size η2 *p* = .06 (medium), and a significance level α = 0.05. To account for potential data loss, 50 participants were recruited for the study (20 males, with an average age of 21.13 ± 0.89 years), all of whom had intermediate English proficiency and had passed the National College English Test at Band 4 in China. One participant with a sentence-judgment task accuracy below 80% was excluded from the analysis (see [28]). Data from 49 participants were ultimately analyzed, with an average accuracy of 95.47% in the sentence judgment, indicating high engagement in the reading task. All participants were right-handed, had normal or corrected-to-normal vision, and completed the eye-tracking reading task. All participants signed an informed consent form in accordance with the Declaration of Helsinki (1991) and received 30 RMB for their participation. All experimental procedures were reviewed and approved by the local ethics committee of Zhejiang International Studies University, Hangzhou, China (no. 2025030508).

### Research design

The study employs a within-subject design, incorporating three levels of memory load (low, medium, high), two language types (L1, L2), and two context types (non-switch, switch). The dependent variables include accuracy and reaction times of the sentence judgment task, as well as eye-tracking indicators across various conditions.

This study employed a dual-task paradigm comprising sentence comprehension judgment (Task 1) and string recognition (Task 2) in the context of natural reading. Task 1 was designed to enhance participants’ active engagement with the experimental sentences, while Task 2 aimed to effectively induce cognitive load. It is noteworthy that in this study, the term “natural reading” refers to the presentation of complete sentences for reading comprehension, contrasting with two less ecologically valid paradigms: studies using isolated code-switched words or phrases without sentential context (e.g [29, 30]), and electrophysiological (EEG) or behavioral studies employing rapid serial visual presentation (RSVP) where words are displayed one at a time at a fixed location (e.g [31, 32]). The presentation of whole sentences allows for parafoveal preview and regressions, which are key features of naturalistic text comprehension [23, 33]. While recognizing that laboratory reading of constructed sentences differs from everyday reading, our paradigm represents a notable advancement toward greater ecological validity compared to prevalent cognitive research focusing on isolated or RSVP-based word-level switching tasks.

### Apparatus

The experiment used the EyeLink 1000 Plus eye tracker and two Dell computers: one for the experimenter and the other for the participant. Experimental stimuli were displayed on a 19-inch Dell monitor with a 1000 Hz sampling frequency. The participant’s monitor had a 144 Hz refresh rate, 1920 × 1080 pixel resolution, and was viewed from 70 cm away with the chin fixed on a headrest. Sentences were presented sequentially in Chinese (Song font) and English (Times New Roman font) in white on a black background, using a 40-point font size (1.08° visual angle) with standard word spacing. Participants engaged in binocular reading, and only data from the right eye was recorded.

The experimental program was designed using E-Prime 2.0 software, which is compatible with the EyeLink 1000 Plus Development Kit. Data extraction was conducted using the system’s built-in software, Data Viewer.

### Materials

#### Sentence materials

Twenty-four nouns with a familiarity score of 4.75 or higher from the Snodgrass & Vanderwart [34] noun picture database, as revised by Zhang and Yang [35], were chosen to construct sentences. Each word was used to create four semantically equivalent and grammatically correct English and Chinese sentences following the structure of “an adverb + a main clause,” with the chosen words as objects in the main clause. The target word was positioned in the middle of the sentences to avoid primacy or recency effects. A set of basic 96-sentence materials was created, including four types: Chinese non-switch context (C-NSC), Chinese switch context (C-SC, English-source to Chinese-target context), English non-switch context (E-NSC), and English switch context (E-SC, Chinese-source to English-target). The sentences had an average length of about 20 characters in Chinese and 12 words in English, with no syntactic or semantic ambiguities. Before the formal experiment, a separate of 24 university students who did not participate in the study assessed the difficulty and readability of the materials on a 5-point scale, identifying unfamiliar words and unclear sentences for potential modification or removal. This pre-testing aimed to ensure simplicity and comprehensibility. The final set of 96 experimental sentences had an average difficulty rating of 1.22 ± 0.68 (1 = very easy, 5 = very difficult) and an average readability rating of 4.37 ± 0.56 (1 = very unreadable, 5 = very readable). To assess participants’ comprehension, one-third of the sentences were designed with content-based judgment questions. An example of the sentence materials is presented in Table 1.

**Table 1:** An example of experimental sentences

Sentence types	Experimental sentences	Judgment questions
C-NSC	吃牛排时, 他把**叉子**从桌上拿了起来^a^。	他用**叉子**吃牛排。(Correct)
C-SC	When eating the steak, he picked up the **叉子** from the table.	He used**叉子**to eat steak. (Correct)
E-NSC	When eating the steak, he picked up the **fork** from the table.	He used **knife** to eat steak. (Incorrect)
E-SC	吃牛排时, 他把**fork**从桌上拿了起来。	他用**knife**吃牛排。(Incorrect)

The Chinese sentence “吃牛排时, 他把叉子从桌上拿了起来” is glossed word-by-word as: *eat steak when*,* he BA fork from table take up*. As described in the *Sentence materials* section, the target word “叉子” (fork) is the *object of the main clause* in both languages. This functional equivalence ensures that the critical comparison is not confounded by word order

The word in bold is the target word

#### Load materials

Memory load, a core concept in working memory, denotes the limited capacity to simultaneously retain 5 to 9 basic pieces of information [12, 36]. This study categorizes memory load conditions by the length of the strings participants are required to memorize. The strings in the experiment comprise randomly generated sequences of one-, two-, and three-digit numbers, interspersed with an equal number of uppercase English letters. The experiment manipulates three string lengths: 2 units (e.g., F7), 4 units (e.g., 2R9F), and 6 units (e.g., 3P1B7S), representing low, medium, and high memory load levels, respectively.

The study includes three memory load conditions (low, medium, and high), with the same set of 96 basic sentences presented once per condition, yielding a total of 288 sentence trials. The materials were organized into 12 blocks, with each memory load condition (low, medium, high) consisting of 4 blocks. Within each block, there were 24 sentences, divided equally among C-NSC, C-SC, E-NSC, and E-SC conditions. The experimental conditions were randomized using a Latin square design, with sentence presentation randomized within each block.

### Procedure

The experimental procedure included the following sequence: A white fixation cross was displayed for 300 ms, followed by a 400-ms blank screen. Subsequently, a string consisting of 2–6 units appeared for 4000 ms, followed by a 400-ms blank screen, and then an experimental sentence was shown for 4000 ms. A sentence judgment task ensued for a maximum of 6000 ms, during which participants were required to indicate correctness by pressing “J” for correct or “F” for incorrect. Participants were then tasked with identifying the previously presented string by choosing between two options, pressing “D” for the left option or “K” for the right. Following their selection, a blank screen was presented for 400 ms before the next trial began. Correct key presses were evenly distributed among participants. Before the experiment, participants were instructed to memorize the initial string, comprehend the subsequent sentence, and recognize the previously presented string.

**Fig. 2:**
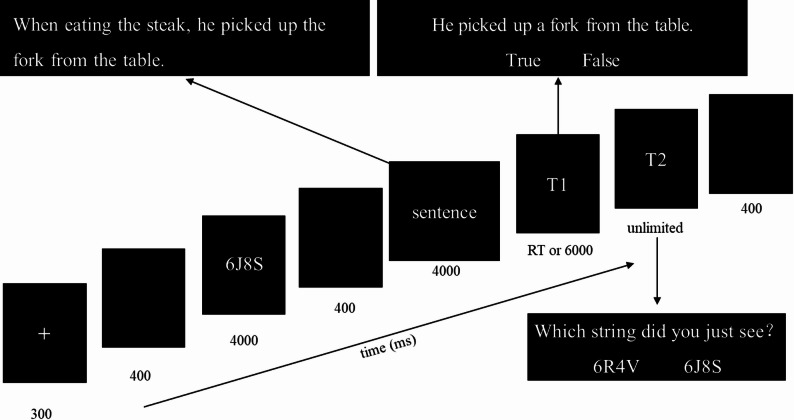
Illustration of one experimental trial

To prevent fatigue during the experiment, participants were instructed to take a 2-minute break after every three blocks and recalibrate the nine-point scale after each break. On average, the experimental session lasted 45 min per participant. The experimental procedure for each trial is depicted in Fig. 2.

### Data analysis

Behavioral data included accuracy (acc.) and reaction times (RTs) in the sentence judgment task. Accuracy is reported as a percentage (%), and RTs are reported in milliseconds (ms). Eye-tracking measures were specifically selected to evaluate various cognitive processes across three dimensions, as illustrated in Fig. 1. Temporal measures (e.g., FFD, GD, RPD) capture the temporal aspects of lexical access and integration; Spatial measures (e.g., RSD, SCpS, FC-TW) represent visual attentional sampling and allocation; Physiological measures (PS, BCpS) indicate cognitive effort and arousal [21, 23]. For eye-tracking analysis, only trials with correct responses in both the sentence judgment task (Task 1) and the subsequent string recognition task (Task 2) were included. This strict criterion ensures that memory load was effectively primed and sentence comprehension was accurately achieved, thereby validating the interpretation of how experimentally induced cognitive load influences language processing. Only under such conditions can statistical findings regarding the modulating role of cognitive load in bilingual switching be considered meaningful.

The eye-tracking data analysis followed the multi-level framework outlined in the Introduction (see Fig. 1). This framework distinguishes between global, sentence-level processing and local, target-word-level processing, with the latter further divided into early-stage (reflecting initial lexical access) and late-stage (reflecting deeper semantic integration and conflict resolution) components. Global analysis included six metrics: fixation duration per sentence (FDpS), rightward saccade distance (RSD), saccade count per sentence (SCpS), fixation count per sentence (FCpS), pupil size (PS), and blink count per sentence (BCpS). Local analysis involved early processing metrics, including first fixation duration (FFD), single fixation duration (SFD), gaze duration (GD), and skipping rate (SR). Late processing metrics included regression path duration (RPD), fixation duration on the target word (FD-TW), regression count (RC), saccade count on the target word (SC-TW), fixation count on the target word (FC-TW), and blink count on the target word (BC-TW).

For all dependent measures, switch effects were calculated to reflect the processing cost associated with language switching. To ensure consistent interpretation across measures, the direction of subtraction was determined by the theoretical meaning of each measure. For measures where larger values indicate greater processing difficulty (e.g., reaction times, fixation durations, gaze durations, regression path durations, fixation counts, saccade counts, pupil size), switch effects were computed as switch context minus non-switch context. A positive value thus represents a switch cost (worse performance in switch trials), and a negative value represents a switch benefit (better performance in switch trials). For measures where larger values indicate better processing efficiency (e.g., accuracy, skipping rate, saccade amplitude), switch effects were computed as non-switch context minus switch context. Again, a positive value represents a switch cost (worse performance in switch trials), and a negative value represents a switch benefit (better performance in switch trials).

To maintain focus on the central findings, detailed results for RSD, FCpS, BCpS, SFD, RC, and SC-TW are reported in the Supplementary Materials. The patterns observed in these measures were consistent with the core findings presented in the Results section.

Data analysis was performed using SPSS 25.0, with a significance level of 0.05. A three-way repeated-measures ANOVA was conducted on behavioral and eye-tracking data, with factors of memory load level (low, medium, high), language type (L1, L2), and context type (non-switch, switch) to explore the impact of memory load on language switching. Furthermore, a two-way repeated-measures ANOVA was utilized with memory load level and switching direction (L1, L2) as factors. In cases of violated sphericity, *p*-values were adjusted using the Greenhouse-Geisser method, and post-hoc tests were conducted using the Bonferroni method to address multiple comparisons.

## Results

The results are structured based on the framework detailed in the Data analysis section. A comprehensive summary of main effects, interactions, and switch effect patterns across all metrics is presented in Table 11, offering a reference for the detailed findings.

### Behavioral data: Acc. and RTs in sentence judgment

**Fig. 3:**
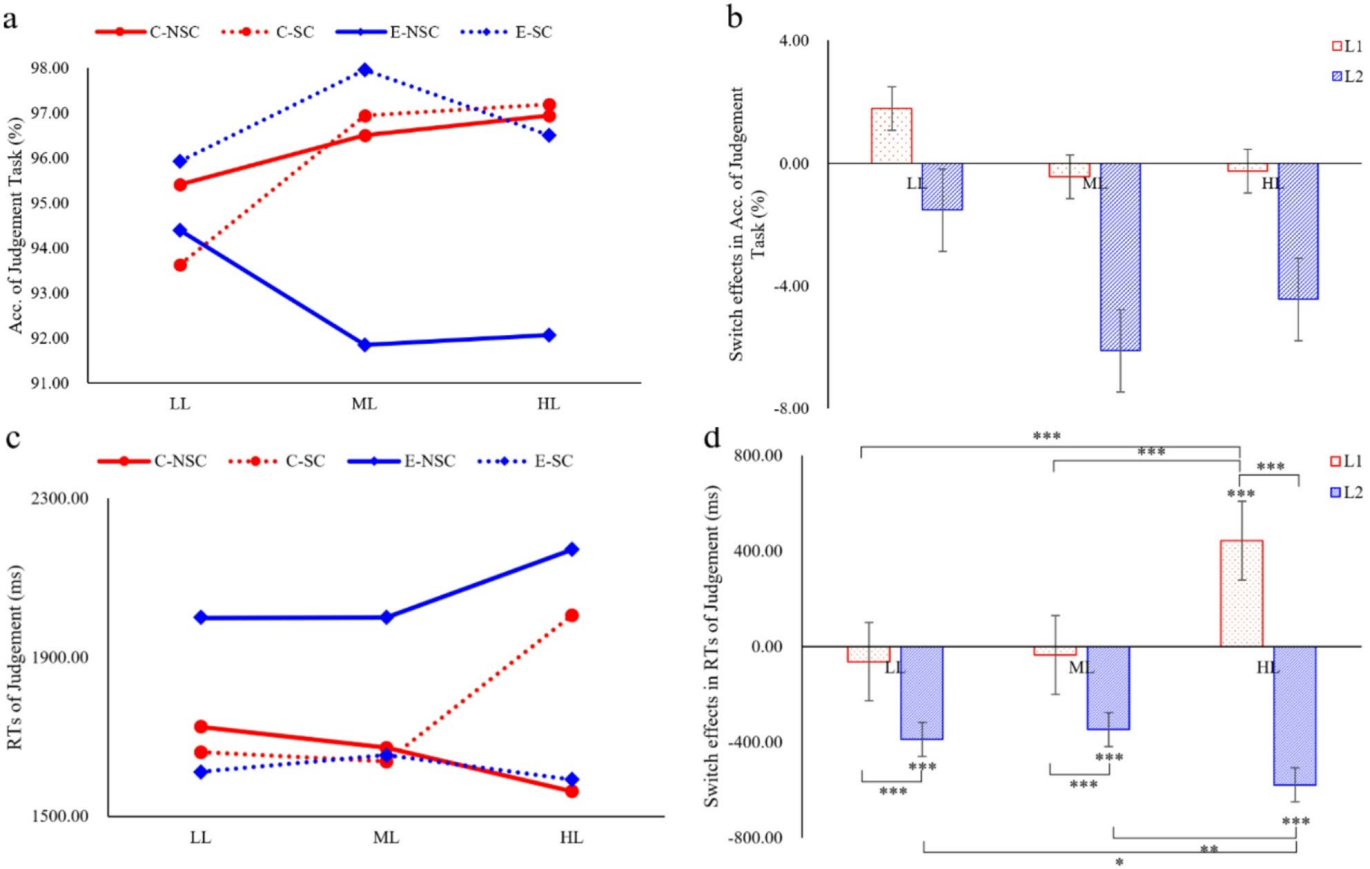
(1) The distribution patterns of Acc. (**a**) and its switch effects (**b**), as well as the RTs (**c**) and their switch effects (**d**) across load levels and language directions; (2) For switch effects, positive values indicate switch costs (lower Acc. or longer RTs in switch trials), and negative values indicate switch benefits (higher Acc. or faster RTs in switch trials); (3) Error bars represent standard errors of the mean. Significance markers above/below bars indicate one-sample *t*-tests against zero (Bonferroni-corrected). Horizontal brackets with asterisks indicate pairwise comparisons in simple effect analyses (Bonferroni-corrected): ***<0.001; **<0.01; *<0.05

One-third of the sentences with judgment tasks were the focus of the accuracy analysis, with 213 trials containing errors (4.53% of the total data) being excluded. In the analysis of reaction time (RT), trials with judgment errors were excluded, as were those with RTs exceeding 3 standard deviations from the mean (6.19% of the total data). Statistical descriptive results for Acc., RTs, and the corresponding switch effects under different conditions are presented in Table 2, with distribution patterns shown in Fig. 3.

**Table 2:** Acc. (%) and RTs (ms) of sentence judgment, as well as their switch effects under various conditions

		Acc.	Acc. effects	RTs	RTs effects
LL	C-NSC	95.41 ± 7.94	1.79 ± 12.24	1725.53 ± 345.87	-63.48 ± 316.16
	C-SC	93.62 ± 8.88		1662.05 ± 385.35	
	E-NSC	94.39 ± 8.86	-1.53 ± 12.11	2000.53 ± 392.25	-388.37 ± 318.83
	E-SC	95.92 ± 7.14		1612.16 ± 379.75	
ML	C-NSC	96.50 ± 8.00	-0.44 ± 8.30	1673.18 ± 361.39	-34.84 ± 320.97
	C-SC	96.94 ± 6.52		1638.34 ± 355.32	
	E-NSC	91.84 ± 9.39	-6.12 ± 9.24	2000.85 ± 434.47	-346.84 ± 333.63
	E-SC	97.96 ± 4.67		1654.01 ± 355.78	
HL	C-NSC	96.94 ± 6.52	-0.25 ± 9.37	1562.89 ± 281.96	443.36 ± 344.08
	C-SC	97.19 ± 5.85		2006.25 ± 478.52	
	E-NSC	92.06 ± 9.89	-4.44 ± 10.92	2171.61 ± 523.23	-578.71 ± 413.5
	E-SC	96.50 ± 8.00		1592.90 ± 310.20	

(1) LL = low load; ML = medium load; HL = high load; (2) Acc. switch effects = Acc. in non-switch context – Acc. in switch context (See Data Analysis for details); RTs effects = RTs in switch context – RTs in non-switch context

A repeated measures ANOVA was conducted on the Acc. of sentence judgment, with factors of load level (low, medium, high), language type (L1, L2), and context type (non-switch, switch). The results showed a non-significant main effect of load (*F* (2, 96) = 0.93, *p* > .05, *η*2 *p* = .02), but significant main effects of language (*F* (1, 48) = 4.90, *p* < .05, *η*2 *p* = .10), and context (*F* (1, 48) = 8.60, *p* < .01, *η*2 *p* = .15). The interaction between load and language was marginally significant (*F* (2, 96) = 3.06, *p* = .052, *η*2 *p* = .06), with higher accuracy in high load for L1 compared to L2 (*p* < .05). The interaction between load and context was significant (*F* (2, 96) = 3.23, *p* < .05, *η*2 *p* = .06), with significantly higher accuracy in switch contexts under medium and high loads compared to non-switch contexts (*ps* < 0.05). Additionally, the interaction between language and context was significant (*F* (1, 48) = 11.07, *p* < .01, *η*2 *p* = .19), with higher accuracy in switch contexts for L2 compared to non-switch contexts (*p* < .001). The three-way interaction among load, language, and context was not significant (*F* (2, 96) = 0.31, *p* > .05, *η*2 *p* = .01).

A repeated-measures ANOVA was conducted on the switch effects in judgment accuracy (calculated as accuracy in non-switch minus switch contexts), with load level (low, medium, high) and switch direction (L1, L2) as within-subject factors. The results revealed significant main effects of load (*F* (2, 96) = 3.23, *p* < .05, *η*2 *p* = .06) and direction (*F* (1, 48) = 11.07, *p* < .01, *η*2 *p* = .19). However, the interaction between the two was not significant (*F* (2, 96) = 0.31, *p* > .05, *η*2 *p* = .01). The significant main effect of direction indicated that the switch effect for L2 was significantly more negative than for L1 (*p* < .01), reflecting a greater accuracy benefit when switching into the nondominant language. Post-hoc comparisons with Bonferroni correction on the load main effect revealed no significant differences between any of the load levels (*ps* > 0.05), indicating that the overall effect of load on accuracy was modest and did not reliably differentiate between individual load conditions.

A repeated-measures analysis of variance was conducted on RTs for sentence judgment, with factors of load, language, and context. The results revealed significant main effects for load (*F* (2, 96) = 12.28, *p* < .001, *η*2 *p* = .20), language (*F* (1, 48) = 61.45, *p* < .001, *η*2 *p* = .56), and context (*F* (1, 48) = 90.82, *p* < .001, *η*2 *p* = .65). Interactions were significant for load and context (*F*(2, 96) = 12.24, *p* < .001, *η*2 *p* = .20), language and context (*F* (1, 48) = 107.78, *p* < .001, *η*2 *p* = .69), and the three-way interaction of load, language, and context (*F* (2, 96) = 26.80, *p* < .001, *η*2 *p* = .36). Simple effect analysis revealed that under high load, RTs for L1 in switch contexts were significantly longer than in non-switch contexts (*p* < .001). Conversely, regardless of load levels, RTs for L2 were shorter in switch contexts than in non-switch contexts (*ps* < 0.001).

A repeated-measures ANOVA was conducted on the switch effects in RTs for sentence judgment, considering load level and switch direction as factors. The analysis showed significant main effects of load (*F* (2, 96) = 12.24, *p* < .001, *η*2 *p* = .20) and direction (*F* (1, 48) = 107.78, *p* < .001, *η*2 *p* = .69), along with an interaction between load and direction (*F* (2, 96) = 26.80, *p* < .001, *η*2 *p* = .36). Simple effect analyses revealed that for direction effects, the switch effect for L2 was significantly more negative than for L1 across all load levels (*ps* < 0.001), indicating higher processing efficiency when switching into the nondominant language. For load effects, for L1, the switch effect was significantly more positive under high load than under low or medium loads (*ps* < 0.001), while for L2, the effect was significantly more negative under high load than under low or medium load (*ps* < 0.05). One-sample *t*-tests revealed that for L1, the effect under high load was significantly positive (*p* < .001 vs.0), indicating a reliable switch cost. Conversely, for L2, all effects were significantly negative across all load levels (*ps* < 0.001 vs. 0), reflecting consistent switch benefits.

### Eye-tracking data

Based on previous studies [37–41], the eye-tracking data exclusion criteria in this study are as follows: (1) eye-tracking failures; (2) fixation durations below 80ms or above 1200ms; (3) sentences with a total fixation frequency per sentence less than 4; (4) data away from ± 3 standard deviations [42]. Overall, 6.2% of invalid data was excluded in sentence analysis, while 4.3% was excluded in target word analysis.

#### Global analysis

##### Temporal dimension: FDpS

**Fig. 4:**
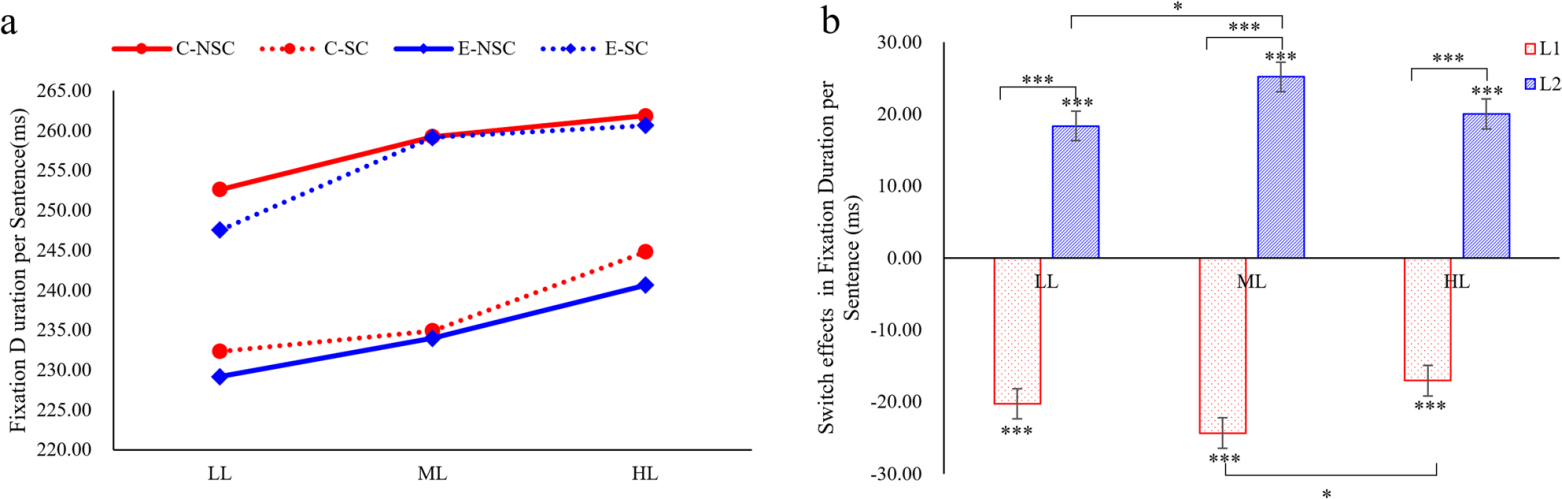
The distribution patterns of FDpS (**a**) and its switch effects (**b**) under various conditions

**Fig. 5:**
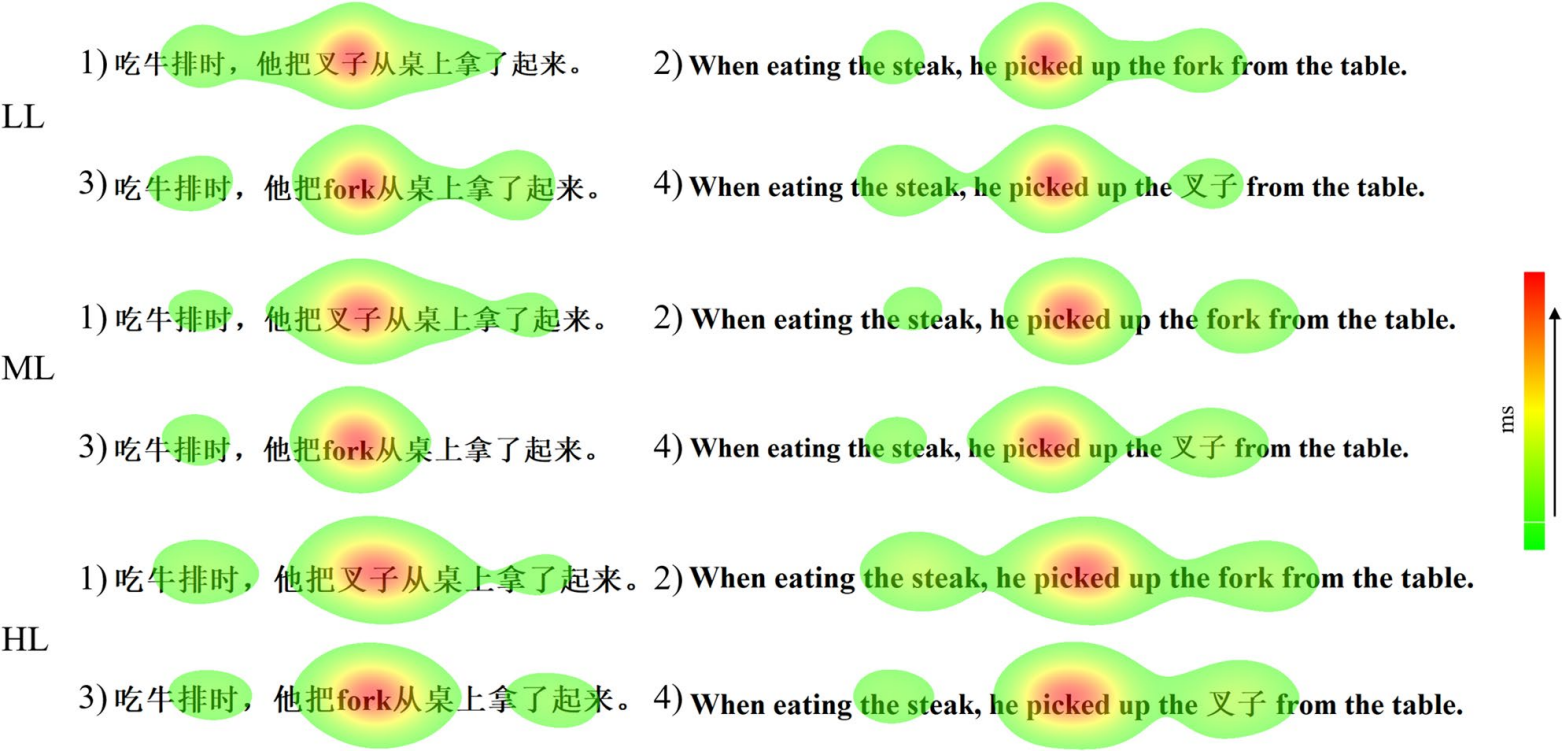
An example of a fixation heatmap for the stimulus “fork/叉子”: (1) C-NSC; (2) E-NSC; (3) C-SC; (4) E-SC

FDpS, representing the mean duration of all fixation points during sentence comprehension, reflects the overall cognitive load of readers [43], with longer duration indicating higher processing demands [44]. Table 3 presents detailed statistical results for FDpS and its switch effects across various conditions, while Fig. 4 illustrates the distribution patterns and Fig. 5 displays the corresponding heatmap.

**Table 3:** FDpS and its switch effects (ms) under various conditions

	C-NSC	C-SC	L1 effects	E-NSC	E-SC	L2 effects
LL	252.62 ± 38.64	232.35 ± 32.38	-20.27 ± 19.72	229.16 ± 29.64	247.53 ± 33.15	18.36 ± 20.76
ML	259.26 ± 40.42	234.93 ± 33.76	-24.34 ± 20.01	233.97 ± 31.42	259.15 ± 38.03	25.18 ± 19.93
HL	261.87 ± 38.6	244.82 ± 41.64	-17.04 ± 24.71	240.64 ± 33.27	260.66 ± 42.05	20.02 ± 19.78

FDpS effects = FDpS in switch context – FDpS in non-switch context

A repeated measures analysis of variance was conducted on FDpS with load, language, and context as factors. The results indicated significant main effects of load (*F* (2, 96) = 21.34, *p* < .001, *η*2 *p* = .31) and language (*F* (1, 48) = 4.29, *p* < .05, *η*2 *p* = .08), but not of context (*F* (1, 48) = 0.07, *p* > .05, *η*2 *p* = .001). Significant interactions were found between language and context (*F* (1, 48) = 103.96, *p* < .001, *η*2 *p* = .68), as well as among load, language, and context (*F* (2, 96) = 6.23, *p* < .01, *η*2 *p* = .12). Further analysis of the three-way interaction revealed longer fixation duration for L1 in non-switch contexts compared to switch contexts, and shorter duration for L2 in non-switch contexts (*ps* < 0.001). The fixation duration per sentence increased with memory load across most conditions. In switch contexts, durations were significantly longer under high load than under low load for both L1 and L2 (*ps* < 0.001). Specifically, in L1 switch contexts, the duration was longest under high load and shortest under low load, with significantly longer duration under high load compared to medium and low loads (*ps* < 0.01). Similarly, in L2 switch contexts, it was longest under high load and shortest under low load, with significantly longer duration under high and medium loads compared to low load (*ps* < 0.001).

A repeated-measures ANOVA on the switch effects for FDpS revealed a non-significant main effect of load (*F* (2, 96) = 0.50, *p* > .05, *η*2 *p* = .01), but a significant main effect of direction (*F* (1, 48) = 103.96, *p* < .001, *η*2 *p* = .68) and a significant interaction between the two (*F* (2, 96) = 6.23, *p* < .01, *η*2 *p* = .12). Simple effect analysis demonstrated that L1 exhibited a significantly more negative impact than L2 across all loads (*ps* < 0.001). Regarding load effects, the switch effect for L1 was notably more negative under medium load compared to high load (*p* < .05), whereas for L2, the effect was significantly more positive under medium load than under low load (*p* < .05). One-sample *t*-tests indicated that for L1, all effects were consistently negative across all load levels (*ps* < 0.001 vs. 0), suggesting reliable switch benefits. In contrast, for L2, all effects were consistently positive across all load levels (*ps* < 0.001 vs. 0), indicating consistent switch costs.

##### Spatial dimensions: SCpS, RSD, and FCpS

**Fig. 6:**
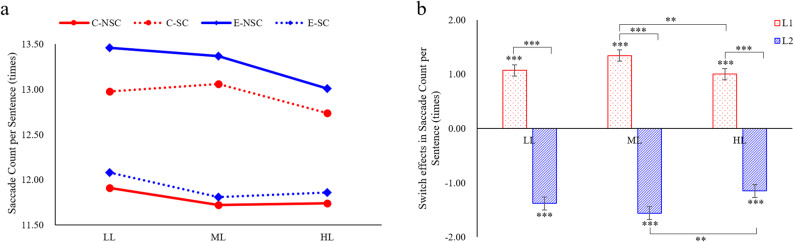
The distribution patterns of SCpS (**a**) and its switch effects (**b**) under various conditions

SCpS represents the frequency of eye movements within a sentence, indicating the interplay between visual sampling strategies and memory processing in reading. A higher eye movement frequency is associated with lower reading fluency, while a lower frequency is linked to automated processing [45]. RSD, the distance between consecutive fixations, is a crucial aspect of eye movements during reading, reflecting readers’ processing efficiency [24]. A larger RSD suggests improved reading efficiency. FCpS denotes the frequency of fixations on the entire sentence, with each fixation typically lasting 200–300 milliseconds to facilitate visual information encoding by the brain. A higher fixation frequency indicates increased cognitive resource involvement [46]. Table 4 presents statistical data on SCpS and the associated switch effects under various conditions, with the corresponding distribution patterns illustrated in Fig. 6. Tables S1 and S2 in the supplementary materials contain the results for RSD and FCpS, including their switch effects, while Figs. S1 and S2 show their distribution patterns, respectively.

**Table 4:** SCpS (times) and the switch effects under various conditions

	C-NSC	C-SC	L1 effects	E-NSC	E-SC	L2 effects
LL	11.91 ± 1.55	12.98 ± 1.77	1.07 ± 1.03	13.46 ± 1.51	12.08 ± 1.46	-1.38 ± 1.12
ML	11.72 ± 1.77	13.06 ± 1.71	1.34 ± 0.98	13.37 ± 1.66	11.81 ± 1.70	-1.56 ± 1.06
HL	11.74 ± 1.76	12.74 ± 2.05	1.00 ± 1.04	13.01 ± 1.63	11.86 ± 1.82	-1.15 ± 1.02

SCpS effects = SCpS in switch context – SCpS in non-switch context

A repeated-measures analysis of variance was conducted on SCpS with the factors load, language, and context. Significant main effects were found for load (*F* (2, 96) = 3.21, *p* < .05, *η*2 *p* = .06), language (*F* (1, 48) = 18.15, *p* < .001, *η*2 *p* = .27), and a marginally significant effect for context (*F* (1, 48) = 3.84, *p* = .056, *η*2 *p* = .07). The interaction between language and context was significant (*F* (1, 48) = 144.58, *p* < .001, *η*2 *p* = .75), along with a three-way interaction among load, language, and context (*F* (2, 96) = 5.94, *p* < .01, *η*2 *p* = .11). Simple analysis of the three-way interaction revealed that saccade frequency was lower for L1 in non-switch context compared to switch context, while for L2, it was higher in non-switch context (*ps* < 0.001). Furthermore, in a non-switch context, saccade frequency was lower for L1 than for L2, but in switch contexts, it was higher for L1 (*ps* < 0.001). Notably, in L1 switch contexts, medium load led to higher saccade frequency than high load (*p* < .05), and in L2 non-switch contexts, medium and low loads resulted in higher saccade frequency compared to high load (*ps* < 0.01). No other interactions between factors reached statistical significance (*ps* > 0.05).

A repeated-measures ANOVA on the switch effects for SCpS revealed a non-significant main effect of load (*F(*2, 96) = 0.28, *p* > .05, *η*2 *p* = .006), but a significant main effect of direction (*F(*1, 48) = 144.58, *p* < .001, *η*2 *p* = .75) and a significant interaction effect (*F(*2, 96) = 5.94, *p* < .01, *η*2 *p* = .11). Simple effect analyses indicated that L2 was significantly more negative than L1 across all loads (*ps* < 0.001) for direction effects. Regarding load effects, the switch effect for L1 was significantly more positive under medium load compared to high load (*p* < .01), whereas for L2, the effect was significantly more negative under medium load than under high load (*p* < .01). One-sample *t*-tests demonstrated that for L1, all effects were significantly positive across all load levels (*ps* < 0.001 vs. 0), indicating consistent switch costs (i.e., more saccades in switch trials). Conversely, for L2, all effects were significantly negative across all load levels (*ps* < 0.001 vs. 0), reflecting consistent switch benefits (i.e., fewer saccades in switch trials).

##### Physiological dimension: PS and BCpS

**Fig. 7:**
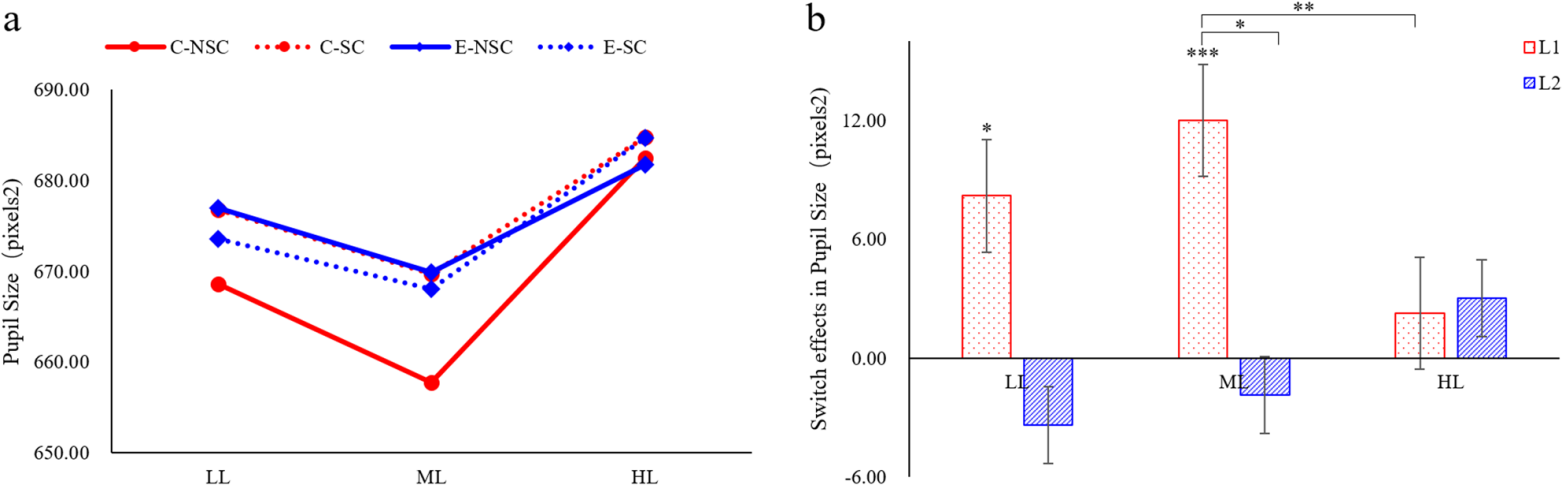
The distribution patterns of PS (**a**) and its switch effects (**b**) under various conditions

PS, a vital physiological measure, reflects rapid cognitive dynamics, capturing cognitive fluctuations at a millisecond level. Pupil dilation, triggered by sympathetic nervous system activation, signifies heightened brain resource allocation and cognitive effort [21]. Pupil area, rather than pupil diameter, offers a more precise evaluation of changes in pupil morphology [47]. BCpS measures blink frequency during sentence reading, thereby enhancing the ecological validity of investigating cognitive neural processes in information processing [25]. Table 5 presents descriptive statistics for PS and switch effects under various conditions, along with their distribution depicted in Fig. 7. Descriptive statistics for BCpS results can be found in Table S3 in the supplementary materials, with the corresponding distribution patterns shown in Fig. S3.

**Table 5:** PS (pixels^2^) and the switch effects under various conditions

	C-NSC	C-SC	L1 effects	E-NSC	E-SC	L2 effects
LL	668.63 ± 179.55	676.81 ± 177.25	8.18 ± 26.97	676.99 ± 174.25	673.61 ± 179.95	-3.38 ± 26.71
ML	657.73 ± 181.34	669.72 ± 181.24	11.99 ± 24.47	669.91 ± 177.90	668.03 ± 182.40	-1.88 ± 23.82
HL	682.51 ± 184.53	684.77 ± 185.40	2.26 ± 19.26	681.72 ± 180.63	684.74 ± 187.98	3.02 ± 22.88

PS effects = PS in switch context – PS in non-switch context

A repeated-measures analysis of variance was conducted on PS with the factors load, language, and context. Significant main effects were found for load (*F* (2, 96) = 9.67, *p* < .001, *η*2 *p* = .17) and context (*F* (1, 48) = 5.61, *p* < .05, *η*2 *p* = .11), while the main effect of language was not significant (*F* (1, 48) = 3.35, *p* > .05, *η*2 *p* = .07). The interaction among load, language, and context was significant (*F* (2, 96) = 3.69, *p* < .05, *η*2 *p* = .07). Simple effect analysis revealed that, in L1 non-switch context, pupil size was largest under high load, smallest under medium load, and significantly different among three loads (*ps* < 0.05), while in L1 switch context, it was larger under high load compared to medium load (*p* < .001). In L2 non-switch context, pupil size was larger under high load compared to medium load (*p* < .01), but in L2 switch context, it was larger under high load compared to low or medium loads (*ps* < 0.05). Furthermore, under low and medium loads, pupil size was significantly smaller in L1 non-switch context compared to L1 switch context (*ps* < 0.05), and under medium load, it was smaller in L1 non-switch context compared to L2 non-switch context (*p* < .01). No significant interactions were observed between other factors (*ps* > 0.05).

A repeated-measures ANOVA on the switch effects for PS showed no significant main effects for load (*F* (2, 96) = 0.78, *p* > .05, *η*2 *p* = .02) or direction (*F* (1, 48) = 3.22, *p* > .05, *η*2 *p* = .06), but a significant interaction between the two (*F* (2, 96) = 3.69, *p* < .05, *η*2 *p* = .07). Simple effect analysis indicated that under medium load, L1 exhibited a significantly more positive direction effect compared to L2 (*p* < .05). Regarding load effects, the switch effect for L1 was significantly more positive under medium load than under high load (*p* < .01), whereas for L2, there was no significant difference in the effect across different loads (*ps* > 0.05). One-sample *t*-tests demonstrated that switch effects for L1 were significantly positive under low and medium loads (*ps* < 0.05 vs. 0), indicating the presence of switch costs. Conversely, for L2, none of the effects significantly deviated from zero across any load conditions (*ps* > 0.05 vs. 0), suggesting the absence of reliable switch effects for the non-dominant language.

#### Local analysis: early processing stage

##### Temporal dimensions: FFD, SFD, and GD

**Fig. 8:**
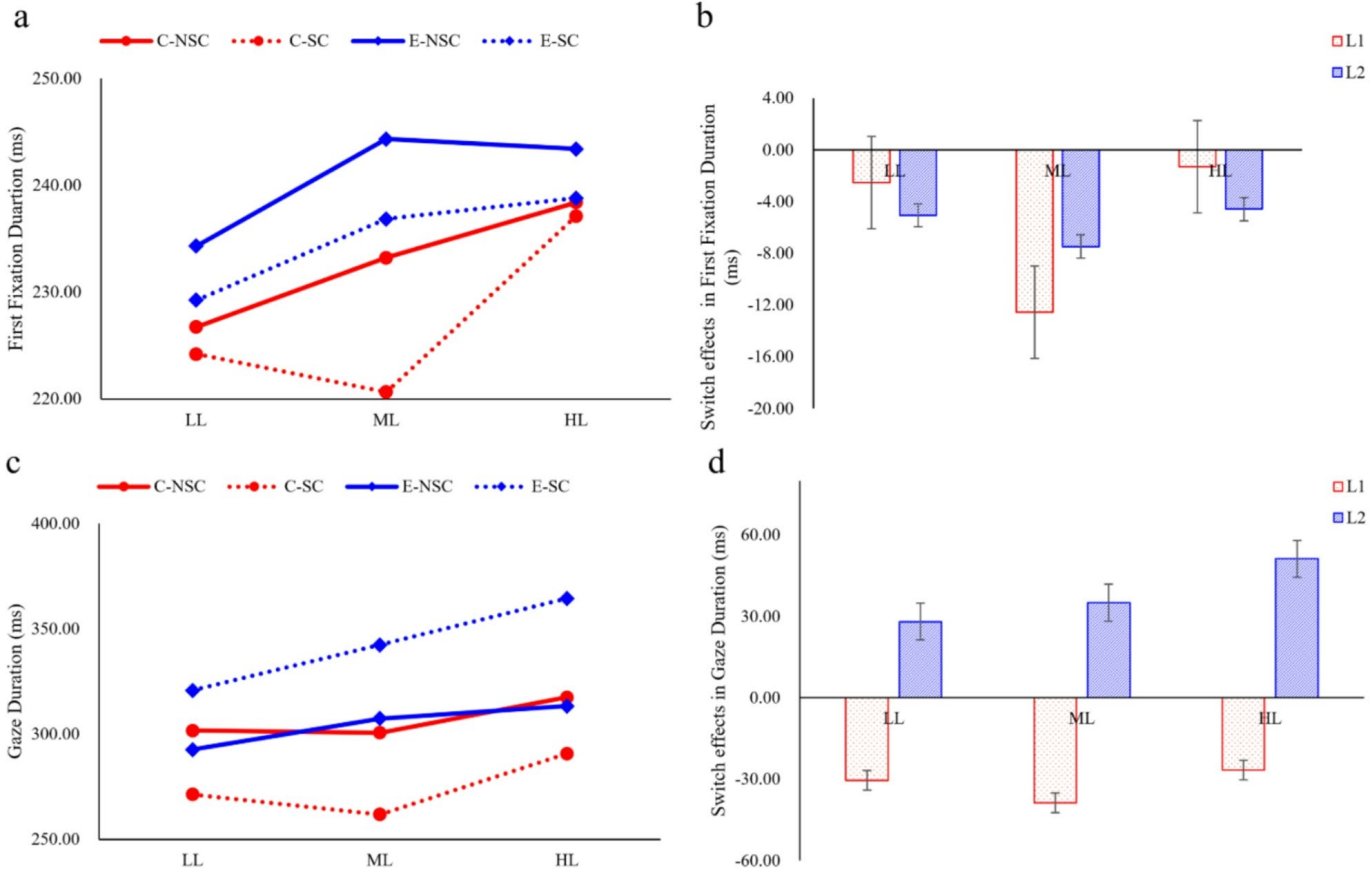
The distribution patterns of FFD (**a**) and its switch effects (**b**), as well as GD (**c**) and its switch effects (**d**) under various conditions

FFD represents the time taken for the initial fixation on a specific area of interest during eye movement, indicating rapid stimulus recognition and early comprehension [48]. A shorter FFD suggests smoother information processing, while a longer FFD may indicate higher memory load, serving as a crucial indicator of early cognitive processing efficiency. SFD refers to the time required for a single fixation within the area of interest during first-pass reading, reflecting the cognitive process of extracting information with a single fixation during initial processing [49]. GD is the total time from the first fixation on a specific area of interest until the eyes move to another area during initial reading. It reflects the readers’ processing and integration of information upon encountering that area for the first time, including vocabulary recognition, semantic activation, and initial contextual integration [50]. A shorter GD typically indicates rapid vocabulary recognition and smooth semantic activation, while a longer GD may suggest challenges in vocabulary processing. Table 6 provides detailed descriptive statistics for FFD and GD, while their distribution patterns are depicted in Fig. 8. The corresponding statistics for SFD are available in Table S4 in the supplementary materials, with its distribution pattern presented in Fig. S4.

**Table 6:** FFD (ms), GD (ms), and their switch effects under various conditions

		FFD	FFD effects	GD	GD effects
LL	C-NSC	226.75 ± 39.13	-2.54 ± 43.46	301.77 ± 163	-30.40 ± 137.27
	C-SC	224.21 ± 38.97		271.37 ± 92.14	
	E-NSC	234.31 ± 34.36	-5.07 ± 30.14	292.60 ± 70.89	28.12 ± 90.72
	E-SC	229.24 ± 32.67		320.72 ± 111.52	
ML	C-NSC	233.23 ± 42.60	-12.57 ± 37.55	300.62 ± 121.00	-38.78 ± 104.72
	C-SC	220.66 ± 42.27		261.84 ± 109.04	
	E-NSC	244.33 ± 34.00	-7.47 ± 39.81	307.46 ± 75.78	34.99 ± 121.33
	E-SC	236.86 ± 45.31		342.45 ± 146.70	
HL	C-NSC	238.41 ± 48.12	-1.29 ± 50.48	317.50 ± 144.29	-26.67 ± 105.55
	C-SC	237.12 ± 53.66		290.83 ± 107.13	
	E-NSC	243.40 ± 41.40	-4.59 ± 42.42	313.28 ± 85.56	51.22 ± 172.79
	E-SC	238.81 ± 44.15		364.50 ± 192.71	

FFD effects = FFD in switch context – FFD in non-switch context; GD effects = GD in switch context – GD in non-switch context

A repeated-measures analysis of variance was conducted on the FFD of the target word, with the factors load, language, and context. The analysis revealed a significant main effect of load (*F(*2, 96) = 6.57, *p* < .01, *η*2 *p* = .12), indicating longer duration under high load and shorter duration under low load, with a significant difference between them (*p* < .01). A significant main effect of language was also observed (*F(*1, 48) = 7.20, *p* < .05, *η*2 *p* = .13), showing longer duration for L2 compared to L1 (*p* < .05). The main effect of context was marginally significant (*F(*1, 48) = 3.90, *p* = .054, *η*2 *p* = .08), with marginally longer duration in non-switch context compared to switch context (*p* = .054). The three-way interaction among load, language, and context was not significant (*F(*2, 96) = 0.31, *p* > .05, *η*2 *p* = .01). No other significant interactions were found (*ps* > 0.05).

A repeated-measures ANOVA on the switch effects for FFD showed no significant main effects of load (*F* (2, 96) = 1.63, *p* > .05, *η*2 *p* = .03) or direction (*F* (1, 48) = 0.002, *p* > .05, *η*2 *p* = .000), and no significant interaction (*F* (2, 96) = 0.31, *p* > .05, *η*2 *p* = .01), indicating that memory load did not reliably modulate first fixation durations during language switching.

A repeated-measures analysis of variance was conducted on the GD of the target word, with the factors load, language, and context. The results indicated a significant main effect of load (*F* (2, 96) = 4.30, *p* < .05, *η*2 *p* = .08), showing longer gaze duration under high load compared to low load, with a significant difference between them (*p* < .05). A significant main effect of language was also observed (*F* (1, 48) = 23.80, *p* < .001, *η*2 *p* = .33), demonstrating longer duration for L2 compared to L1 (*p* < .001). However, the main effect of context was not significant (*F* (1, 48) = 0.25, *p* > .05, *η*2 *p* = .01). An interaction between language and context was significant (*F* (1, 48) = 6.73, *p* < .05, *η*2 *p* = .12), revealing that for L1, gaze duration was longer in non-switch context than in switch context (*p* < .05), while for L2, it was shorter under non-switch contexts (*p* < .05). Additionally, in switch contexts, gaze duration for L1 was shorter than for L2 (*p* < .001). The three-way interaction among load, language, and context was not significant (*F* (2, 96) = 0.41, *p* > .05, *η*2 *p* = .01). No other interactions were found to be significant (*ps* > 0.05).

A repeated-measures ANOVA on the GD switch effects revealed a non-significant main effect of load (*F* (2, 96) = 0.75, *p* > .05, *η*2 *p* = .02), a significant main effect of direction (*F* (1, 48) = 6.73, *p* < .05, *η*2 *p* = .12), and a non-significant interaction (*F*(2, 96) = 0.41, *p* > .05, *η*2 *p* = .01). The significant main effect of direction showed that the switch effect for L1 was significantly more negative than for L2 (*p* < .05).

##### Spatial dimension: SR

**Fig. 9:**
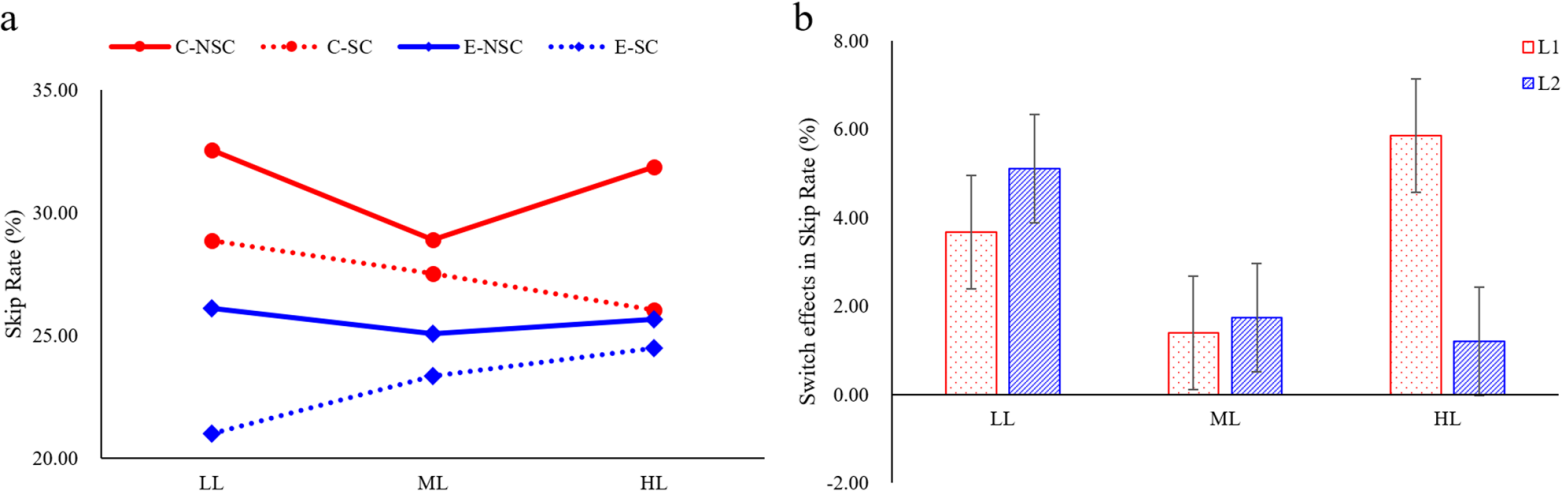
The distribution patterns of SR (**a**) and its switch effects (**b**) under various conditions

SR refers to the likelihood of skipping over target words without pausing, revealing the dynamic processing framework known as the “preview-select-integrate” model in human reading [51]. According to this model, readers do not decode words sequentially; instead, they use parafoveal preview to obtain upcoming information and make strategic processing decisions based on their current cognitive state, deciding whether to process a specific area in detail. SR serves as a key indicator of reading efficiency. Detailed statistics regarding SR for target words and the switch effects under various conditions are provided in Table 7, while the distribution pattern in Fig. 9.

**Table 7:** SR (%) and its switch effects under various conditions

	C-NSC	C-SC	L1 effects	E-NSC	E-SC	L2 effects
LL	32.57 ± 13.49	28.89 ± 16.08	3.68 ± 17.17	26.11 ± 15.28	21.01 ± 10.89	5.10 ± 14.89
ML	28.91 ± 10.29	27.52 ± 17.77	1.39 ± 20.25	25.09 ± 15.51	23.35 ± 11.40	1.74 ± 16.69
HL	31.89 ± 10.91	26.04 ± 16.85	5.85 ± 17.73	25.68 ± 16.39	24.48 ± 12.23	1.20 ± 16.43

SR switch effects = SR in non-switch context – SR in switch context (See Data Analysis for details)

A repeated measures analysis of variance was conducted on the SR of target words, with factors of load, language, and context. The results indicated a non-significant main effect of load (*F* (2, 96) = 0.60, *p* > .05, *η*2 *p* = .01). However, a significant main effect of language was observed (*F* (1, 48) = 18.71, *p* < .001, *η*2 *p* = .28), showing a notably higher skipping rate for L1 compared to L2 (*p* < .001). Additionally, a significant main effect of context was found (*F* (1, 48) = 8.34, *p* < .01, *η*2 *p* = .15), revealing that the skipping rate in non-switch context was significantly higher than in switch context (*p* < .01). The interaction among load, language, and context was not significant (*F* (2, 96) = 1.40, *p* > .05, *η*2 *p* = .03). No other significant interactions were found (*ps* > 0.05).

A repeated-measures ANOVA on the switch effects for SR (calculated as non-switch minus switch) revealed no significant main effects of load (*F(*2, 96) = 1.82, *p* > .05, *η*2 *p* = .04) or direction (*F(*1, 48) = 0.08, *p* > .05, *η*2 *p* = .002), and no significant interaction (*F(*2, 96) = 1.40, *p* > .05, *η*2 *p* = .03). This suggests that memory load did not reliably modulate skipping rates during language switching.

#### Local analysis: late processing stage

##### Temporal dimension: RPD and FD-TW

**Fig. 10:**
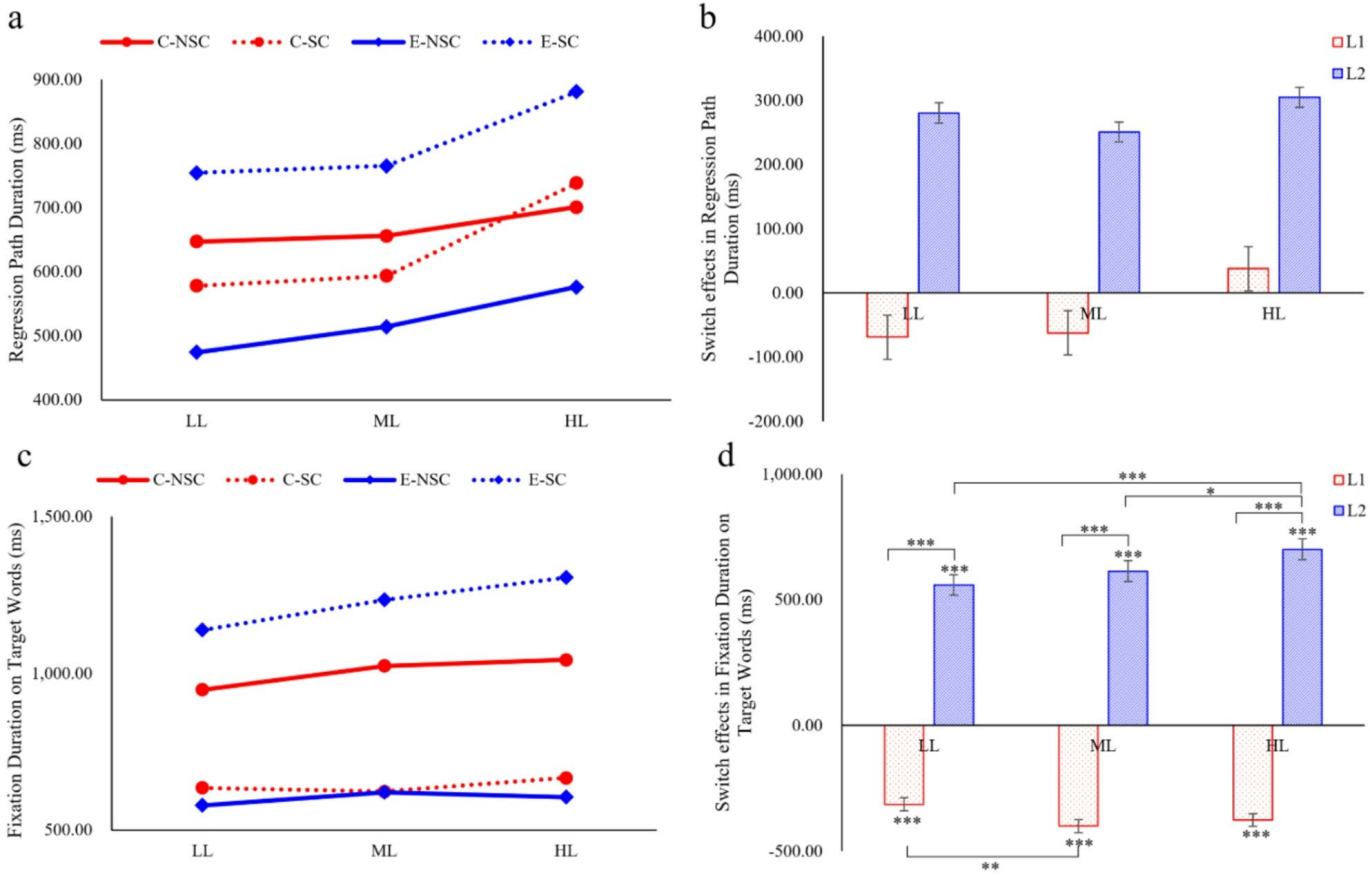
The distribution patterns of RPD (**a**) and its switch effects (**b**), as well as FD-TW (**c**) and its effects (**d**) under various conditions

RPD represents the cumulative time spent on regressions from the initial fixation on the target word until the earliest rightward movement away from that region, reflecting the need for rereading or information integration. Unlike measures of early lexical access, regressions capture the process in which readers actively revisit previous text to address semantic integration challenges following the initial processing [52]. FD-TW signifies the total duration of all fixations on a specific target word, indicating the overall complexity of lexical processing and allocation of cognitive resources. Prolonged fixation duration indicates more intricate lexical access or semantic integration processes [53]. Detailed statistical results for RPD, FD-TW, and their switch effects under various conditions are provided in Table 8, with the distribution pattern illustrated in Fig. 10.

**Table 8:** RPD (ms), FD-TW (ms), and their switch effects under various conditions

		RPD	RPD effects	FD-TW	FD-TW effects
LL	C-NSC	647.19 ± 269.66	-68.96 ± 287.12	948.15 ± 321.77	-313.58 ± 282.71
	C-SC	578.23 ± 242.45		634.57 ± 258.06	
	E-NSC	474.26 ± 214.43	280.36 ± 270.66	579.12 ± 208.24	559.12 ± 272.31
	E-SC	754.62 ± 250.17		1138.24 ± 321.21	
ML	C-NSC	655.91 ± 194.56	-62.19 ± 316.35	1023.39 ± 301.90	-400.11 ± 240.62
	C-SC	593.72 ± 316.06		623.28 ± 262.86	
	E-NSC	514.44 ± 192.33	250.83 ± 247.76	620.86 ± 222.01	613.67 ± 252.34
	E-SC	765.27 ± 260.87		1234.53 ± 361.06	
HL	C-NSC	700.99 ± 221.59	37.73 ± 408.27	1042.96 ± 280.59	-376.26 ± 238.80
	C-SC	738.72 ± 414.39		666.70 ± 312.52	
	E-NSC	576.40 ± 243.93	304.93 ± 273.47	605.15 ± 195.06	700.91 ± 313.35
	E-SC	881.33 ± 313.97		1306.06 ± 394.66	

RPD effects = RPD in switch context – RPD in non-switch context; FD-TW effects = FD-TW in switch context – FD-TW in non-switch context

A repeated-measures analysis of variance was conducted on the RPD of the target word, with the factors load, language, and context. The results indicated a significant main effect of load (*F* (2, 96) = 25.21, *p* < .001, *η*2 *p* = .34). In contrast, the main effect of language was not significant (*F* (1, 48) = 0.27, *p* > .05, *η*2 *p* = .01). However, a significant main effect of context was observed (*F* (1, 48) = 34.14, *p* < .001, *η*2 *p* = .42), along with a significant interaction between load and context (*F* (2, 96) = 3.75, *p* < .05, *η*2 *p* = .07). Subsequent analysis revealed that the regression duration was longer under high load compared to medium or low loads (*ps* < 0.01), regardless of context. It was significantly longer in the switch context than in the non-switch context (*ps* < 0.01), regardless of load condition. Additionally, a significant interaction between language and context was also found (*F* (1, 48) = 27.04, *p* < .001, *η*2 *p* = .36), with regression duration being longer for L1 than for L2 under non-switch context but significantly shorter for L1 than for L2 under switch context (*ps* < 0.001). However, the three-way interaction among load, language, and context was not significant (*F* (2, 96) = 1.10, *p* > .05, *η*2 *p* = .02), and no other interactions were significant (*ps* > 0.05).

A repeated-measures ANOVA on the switch effects for RPD indicated significant main effects of load (*F* (2, 96) = 3.75, *p* < .05, *η*2 *p* = .07) and direction (*F* (1, 48) = 27.04, *p* < .001, *η*2 *p* = .36). However, the interaction was not significant (*F* (2, 96) = 1.10, *p* > .05, *η*2 *p* = .02). The significant main effect of direction showed that the switch effect for L1 was significantly more negative than for L2 (*p* < .001). Post-hoc analyses indicated that the switch effect, aggregated across languages, exhibited a significantly more positive trend under high load compared to medium load (*p* < .05).

A repeated-measures analysis of variance was performed on FD-TW, with the factors load, language, and context. The results demonstrated significant main effects of load (*F* (2, 96) = 18.57, *p* < .001, *η*2 *p* = .28), language (*F* (1, 48) = 38.99, *p* < .001, *η*2 *p* = .45), and context (*F* (1, 48) = 56.18, *p* < .001, *η*2 *p* = .54). Significant interactions were observed between load and context (*F* (2, 96) = 3.22, *p* < .05, *η*2 *p* = .06), language and context (*F* (1, 48) = 291.53, *p* < .001, *η*2 *p* = .86), as well as load, language, and context (*F* (2, 96) = 11.72, *p* < .001, *η*2 *p* = .20). Analysis of the three-way interaction revealed that fixation duration on the target word for L1 was longer in non-switch context compared to switch context, regardless of load conditions, while for L2, the duration was shorter in non-switch context (*ps* < 0.001). Moreover, in a non-switch context, the duration for L1 exceeded that for L2, whereas in a switch context, the duration for L1 was shorter. Additionally, in non-switch context for L1, duration significantly increased under high and medium loads compared to low load (*ps* < 0.01), whereas in switch context, duration was significantly longer under high load compared to medium load (*p* < .05). For L2 in non-switch context, duration was significantly longer under medium load compared to low load (*p* < .05), while in switch context, duration significantly increased under high load compared to low and medium loads (*ps* < 0.05).

A repeated-measures ANOVA on the switch effects for FD-TW revealed significant main effects of load (*F(*2, 96) = 3.22, *p* < .05, *η*2 *p* = .06) and direction (*F(*1, 48) = 291.53, *p* < .001, *η*2 *p* = .86), as well as a significant interaction (*F(*2, 96) = 11.72, *p* < .001, *η*2 *p* = .20). Simple effect analysis showed that L1 was significantly more negative than L2 across all loads (*ps* < 0.001). For load effects, the switch effect for L1 was significantly more negative under medium load than under low load (*p* < .01), while for L2, the effect was significantly more positive under high load than under medium and low load (*ps* < 0.05). One-sample *t*-tests revealed that for L1, the effects were significantly negative across all load levels (*ps* < 0.001 vs. 0), indicating switch benefits. Conversely, for L2, all effects were significantly positive (*ps* < 0.001 vs. 0), reflecting consistent switch costs.

##### Spatial dimension: RC, SC-TW, and FC-TW

**Fig. 11:**
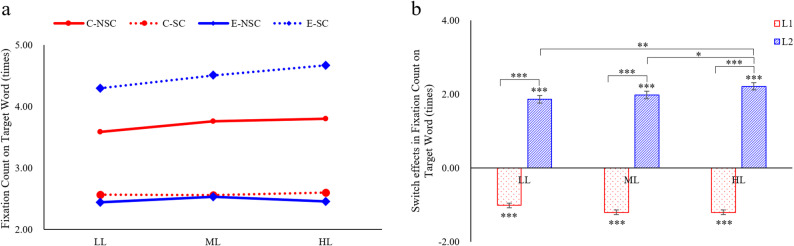
The distribution patterns of FC-TW (**a**) and its switch effects (**b**) under various conditions

RC denotes the frequency of regression count due to revisits to a target word following the initial encounter, indicating challenges in word processing and the need to reconstruct its representation [54]. SC-TW signifies the saccade count of eye movements between the current fixation point and the target word [55]. A higher frequency of saccades implies the requirement for multiple relocations to assimilate information, while a lower frequency indicates efficient information extraction through single or brief fixations. FC-TW, a fundamental metric for gauging the depth of language processing, quantifies the frequency of fixations on the target word and serves as a checkpoint for late-stage processing [56]. A higher frequency of fixations suggests an increased reliance on repeated processing for information access, reflecting an objective assessment of comprehension difficulty and cognitive resource allocation. Table 9 displays the descriptive results of FC-TW, while its distribution patterns are illustrated in Fig. 11. The results for RC and SC-TW can be found in Tables S5 and S6 in the supplementary materials, with their respective distribution patterns shown in Figs. S5 and S6.

**Table 9:** FC-TW (times), and the switch effects under various conditions

	C-NSC	C-SC	L1 effects	E-NSC	E-SC	L2 effects
LL	3.59 ± 0.83	2.57 ± 0.68	-1.01 ± 0.84	2.44 ± 0.64	4.30 ± 0.87	1.86 ± 0.74
ML	3.76 ± 0.79	2.56 ± 0.73	-1.20 ± 0.77	2.53 ± 0.69	4.51 ± 0.92	1.98 ± 0.75
HL	3.80 ± 0.73	2.60 ± 0.88	-1.20 ± 0.76	2.46 ± 0.53	4.67 ± 0.95	2.21 ± 0.84

FC-TW effects = FC-TW in switch context – FC-TW in non-switch context

A repeated-measures analysis of variance was conducted on FC-TW, with load, language, and context as factors. The results revealed significant main effects of load (*F* (2, 96) = 6.76, *p* < .01, *η*2 *p* = .12), language (*F* (1, 48) = 70.92, *p* < .001, *η*2 *p* = .60), and context (*F* (1, 48) = 95.68, *p* < .001, *η*2 *p* = .67). A significant interaction was found between language and context (*F* (1, 48) = 340.67, *p* < .001, *η*2 *p* = .88), as well as a significant three-way interaction among load, language, and context (*F* (2, 96) = 7.79, *p* < .01, *η*2 *p* = .14). Further analysis revealed that, regardless of load conditions, fixation frequency was significantly higher for L1 in non-switch context compared to switch context, but was lower for L2 in non-switch context (*ps* < 0.001). Fixation frequency was higher for L1 than for L2 in a non-switch context, but lower for L1 in a switch context (*ps* < 0.001). In the non-switch context for L1 and the switch context for L2, fixation frequency was higher under medium and high loads compared to low load (*ps* < 0.05). No other interactions among factors reached significance (*ps* > 0.05).

A repeated-measures ANOVA on the switch effects for FC-TW revealed a non-significant main effect of load (*F* (2, 96) = 1.32, *p* > .05, *η*2 *p* = .03), a significant main effect of direction (*F* (1, 48) = 340.67, *p* < .001, *η*2 *p* = .88), and a significant interaction (*F* (2, 96) = 7.79, *p* < .01, *η*2 *p* = .14). Simple effect analysis demonstrated that, in terms of direction effects, L1 exhibited significantly more negativity than L2 across all loads (*ps* < 0.001). Regarding load effects, for L1, the switch effect did not exhibit significant differences across different loads (*ps* > 0.05), whereas for L2, the effect was notably more positive under high load compared to medium and low load (*ps* < 0.05). One-sample *t*-tests indicated that for L1, the effects were consistently negative across all loads (*ps* < 0.001 vs. 0), suggesting reliable switch benefits. In contrast, for L2, the effects were consistently positive across all loads (*ps* < 0.001 vs. 0), indicating persistent switch costs.

##### Physiological dimension: BC-TW

**Fig. 12:**
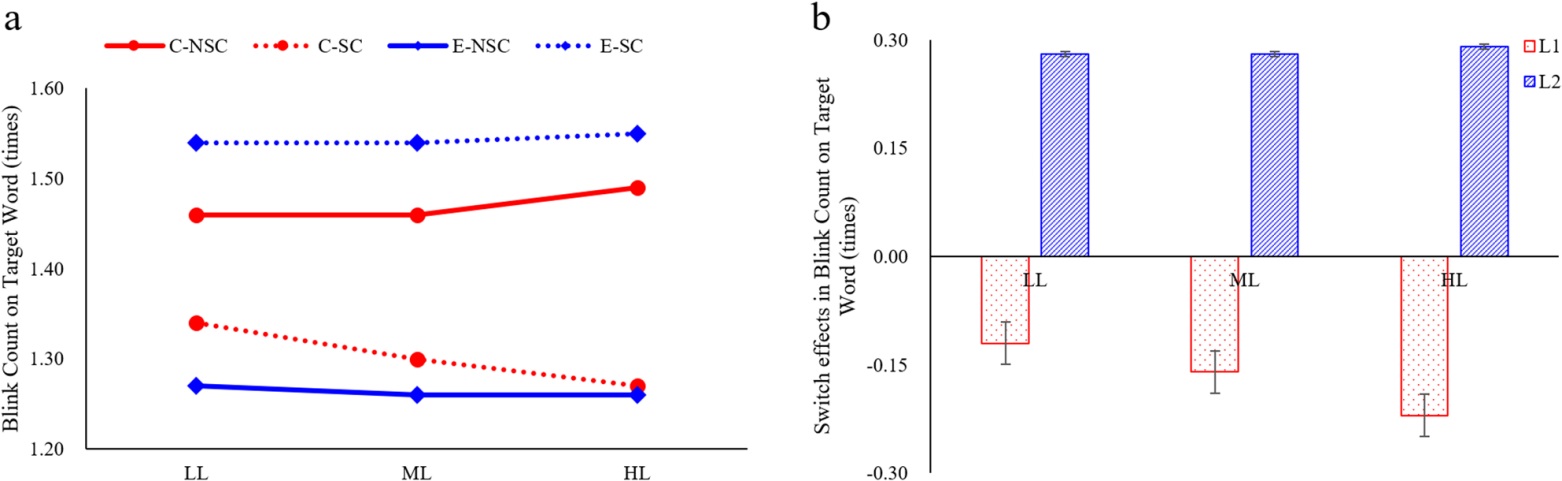
The distribution patterns of BC-TW (**a**) and its switch effects (**b**) under various conditions

BC-TW denotes the frequency of complete eyelid closures and reopenings during fixation on a specific word. Blinks lasting over 50 ms are considered valid, serving as a biomarker for memory load and fatigue as well as a rhythmic cue for information processing [57]. Statistical findings on blink frequency for target words and the switch effects under different conditions are detailed in Table 10, with distribution patterns depicted in Fig. 12.

**Table 10:** BC-TW (times) and its switch effects under various conditions

	C-NSC	C-SC	L1 effects	E-NSC	E-SC	L2 effects
LL	1.46 ± 0.48	1.34 ± 0.39	-0.12 ± 0.31	1.27 ± 0.24	1.54 ± 0.48	0.28 ± 0.41
ML	1.46 ± 0.42	1.3 ± 0.31	-0.16 ± 0.28	1.26 ± 0.26	1.54 ± 0.49	0.28 ± 0.37
HL	1.49 ± 0.47	1.27 ± 0.26	-0.22 ± 0.36	1.26 ± 0.29	1.55 ± 0.58	0.29 ± 0.41

BC-TW effects = BC-TW in switch context – BC-TW in non-switch context

A repeated-measures analysis of variance was conducted on BC-TW with the factors load, language, and context. The results showed that the main effects of load (*F* (2, 96) = 0.76, *p* > .05, *η*2 *p* = .01) and language (*F* (1, 48) = 1.69, *p* > .05, *η*2 *p* = .03) were not significant. However, the main effect of context was significant (*F* (1, 48) = 12.43, *p* < .01, *η*2 *p* = .21). Moreover, there was a significant interaction between language and context (*F* (1, 48) = 33.84, *p* < .001, *η*2 *p* = .41), indicating more blinks for L1 in non-switch context than in switch context (*p* < .001). Instead, fewer blinks for L2 in non-switch context (*p* < .001). Blinks were significantly more frequent for L1 than for L2 in the non-switch context (*p* < .001), while in the switch context, blinks were significantly fewer for L1 (*p* < .001). The three-way interaction among load, language, and context was not significant (*F* (2, 96) = 1.26, *p* > .05, *η*2 *p* = .03). All other interactions were not significant (*ps* > 0.05).

A repeated-measures ANOVA on the switch effects for BC-TW revealed a non-significant main effect of load (*F* (2, 96) = 0.77, *p* > .05, *η*2 *p* = .02), a significant main effect of direction (*F* (1, 48) = 33.84, *p* < .001, *η*2 *p* = .41), and a non-significant interaction (*F* (2, 96) = 1.26, *p* > .05, *η*2 *p* = .03). The significant main effect of direction showed that L1 displayed a more negative trend than L2 (*p* < .001).

### A summary of the modulatory effects of memory load at various levels and stages

Table 11 summarizes the main effects of load, language, and context, as well as the language-context interaction and the three-way interaction among them, as well as significant switch effects.

**Table 11:** Summary of statistical effects and directional comparisons across various conditions

		Main effects			Interaction effects		Comparison
		Load	Language	Context	Language*Context	Load*Language*Context	L1 vs. L2
Behavioral	Acc.	---	*	**	**	---	L2(-)
	RTs.	***	***	***	***	***	L2(-)
Global	FDpS	***	*	---	***	**	L2(+)
	RSD	---	***	***	---	---	---
	SCpS	*	***	*	***	**	L2(-)
	FCpS	---	***	**	***	*	L2(-)
	PS	***	---	*	---	*	L1(+)
	BCpS	*	**	---	***	---	L2(+)
Local (early)	FFD	**	*	*	---	---	---
	SFD	*	**	---	***	---	L2(+)
	GD	*	***	---	*	---	L2(+)
	SR	---	***	**	---	---	---
Local (late)	RPD	***	---	***	***	---	L2(+)
	FD-TW	***	***	***	***	***	L2(+)
	RC	---	---	---	***	*	L1(-)
	SC-TW	**	***	***	***	**	L2(+)
	FC-TW	**	***	***	***	**	L2(+)
	BC-TW	---	---	**	***	---	L2(+)

1) Global processing refers to sentence-level measures; Local processing refers to target-word-level measures, subdivided into early and late stages; 2) The “Directional comparison (L1 vs. L2)” column presents the outcomes of paired-sample t-test analyses that compare the switch effects of L1 and L2. “L1 (−)” denotes that L1 was significantly more negative than L2, while “L1 (+)” indicates that L1 was significantly more positive than L2. Similarly, “L2 (−)” and “L2 (+)” are interpreted in the same manner. A blank entry (---) signifies no significant directional distinction. For the absolute nature (cost vs. benefit) of each measure, refer to the one-sample t-tests against zero in the respective results sections. Asterisks indicate significance levels of main effects or interactions: *** < 0.001; ** < 0.01; * < 0.05

The effects observed in Acc, RTs, FDpS, SCpS, PS, and BCpS during global sentence processing, and in FFD, SFD, and GD during early target word processing, as well as SC-TW and FC-TW during late target word processing, emphasize the significant role of memory load on bilingual reading processes across various levels and processing stages. This suggests that variations in memory load have a substantial impact on multiple aspects of sentence processing, including total processing time, eye movement patterns, cognitive resource allocation, and attention maintenance. Differences in memory load notably affect the initial perceptual encoding and recognition efficiency of target words during the early stages of processing. In the later stage, memory load continues to play a significant regulatory role in higher-level processes such as deep semantic integration and information reassessment for target words.

The impact of memory load on language switching manifested in distinct patterns across measures. In global sentence processing, the temporal measure (FDpS) showed that L2 consistently exhibited switch costs (longer fixations in switch trials), whereas L1 exhibited switch benefits (shorter fixations in switch trials). In contrast, the spatial measures revealed the opposite asymmetry: L1 showed switch costs (more saccades in SCpS; more fixations in FCpS) while L2 showed switch benefits (fewer saccades and fixations). For the physiological measure PS, L1 showed switch costs under low and medium load, whereas L2 showed no reliable switch effects. In local early processing, temporal measures (FFD, GD) showed no reliable switch effects for either language, whereas the spatial measure SR also showed no reliable effects. In local late processing, the temporal measures (RPD, FD-TW) revealed a clear pattern: L1 exhibited switch benefits (fewer regressions and shorter fixation times) across all load levels, while L2 exhibited switch costs (more regressions and longer fixation times) across all load levels. The spatial measure FC-TW mirrored this asymmetry: L1 showed switch benefits (fewer fixations) and L2 showed switch costs (more fixations). The physiological measure BC-TW showed that L1 exhibited switch benefits (fewer blinks) whereas L2 exhibited switch costs (more blinks). Taken together, these findings reveal a systematic dissociation: the nondominant language (L2) bore temporal and attentional costs across most processing stages, while the dominant language (L1) benefited temporally but incurred spatial costs, a pattern that underscores the distinct roles of automaticity and controlled processing in bilingual comprehension under varying memory load.

## Discussion

This study employed a multi-level eye-tracking framework to dissect the interplay between working memory load and language switching during bilingual sentence comprehension. Our findings reveal that this interplay is not monolithic but is dynamically shaped by the interactions among cognitive resources, task demands, and processing stages.

### The triple interaction of memory load, language, and context

**Fig. 13:**
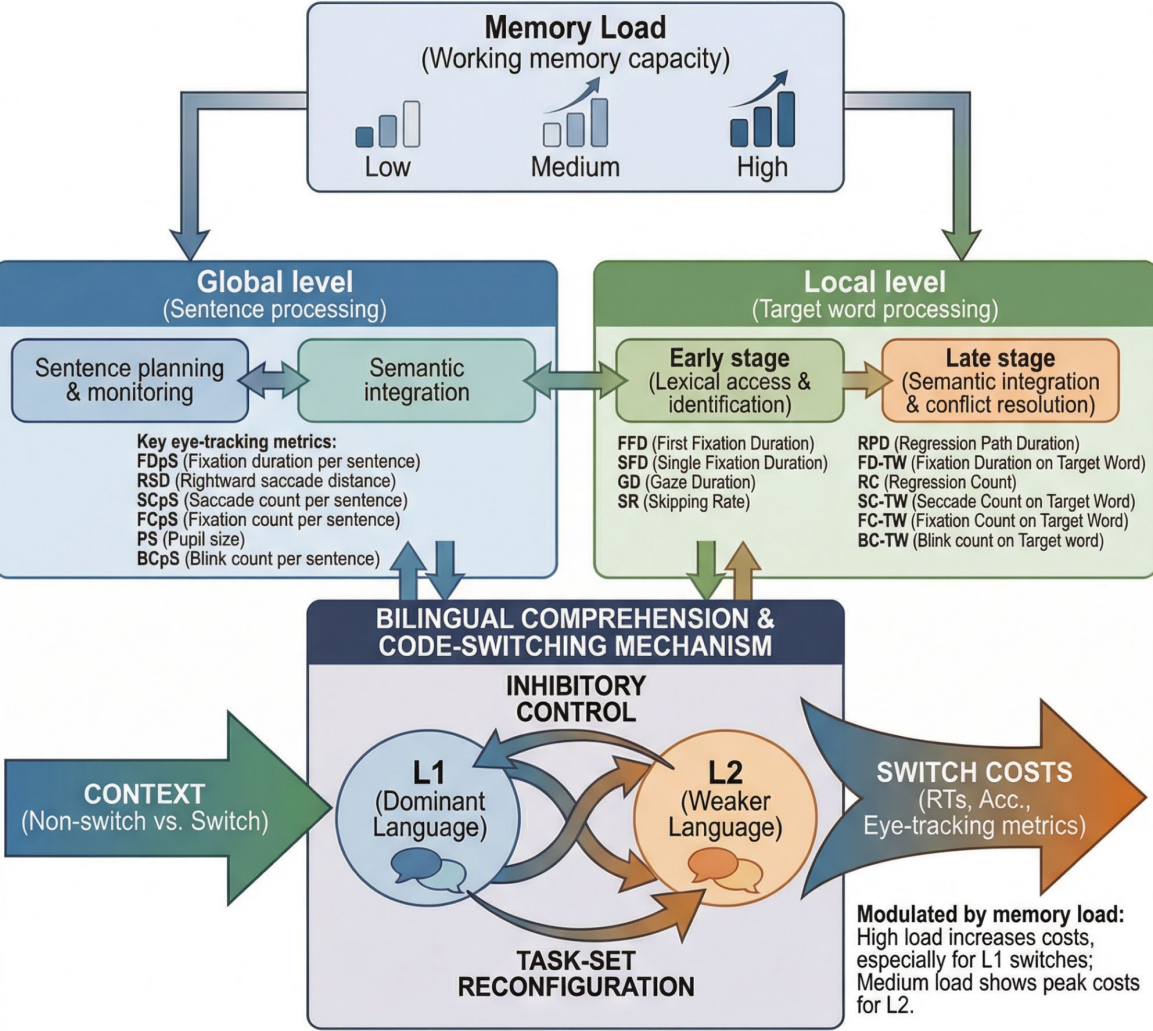
Language switching mechanism in bilingual comprehension under memory load

The central and most consistent finding across our data is a significant three-way interaction among memory load, language type, and context type. This interaction was not a uniform amplification of difficulty but manifested in distinct, level-specific patterns, providing multifaceted evidence for the Adaptive Control Hypothesis [11] (see Fig. 13). Crucially, the nature of this interaction differed between global sentence-level processing and local word-level processing.

At the sentence level, the triple interaction revealed a dissociation between temporal and spatial processing dimensions under cognitive load. Temporally, FDpS showed that L1 consistently exhibited switch benefits (shorter fixations in switch trials) while L2 consistently exhibited switch costs (longer fixations in switch trials). This pattern suggests that switching into the dominant language facilitates global reading fluency, whereas switching into the nondominant language imposes a temporal cost. Spatially, by contrast, SCpS and FCpS revealed the opposite pattern: L1 showed consistent switch costs (more saccades/fixations in switch trials) while L2 showed consistent switch benefits (fewer saccades/fixations in switch trials). This indicates that the dominant language incurs a spatial cost when switched into, whereas the nondominant language benefits spatially from a switch. Moreover, load exerted selective modulations within these dimensions. For L1, the temporal benefit was most pronounced under medium load, whereas the spatial cost was also largest under medium load. For L2, the temporal cost was largest under medium load, whereas the spatial benefit was also largest under medium load. This suggests that the allocation of limited cognitive resources differentially affects temporal and spatial aspects of oculomotor control, with medium load perhaps maximizing competition between language control and semantic integration (reflected in the temporal cost for L2), while simultaneously eliciting the most effective spatial reallocation strategy (reflected in the spatial benefit for L2). Under high load, the spatial benefit for L2 diminished significantly, whereas the spatial cost for L1 remained significant but was smaller than under medium load. This pattern suggests that when cognitive resources are severely depleted, the system may prioritize maintaining temporal efficiency at the expense of spatial optimization, with the spatial dimension, particularly proactive planning mechanisms, showing greater vulnerability to resource constraints. This early divergence between how load affects *when* we look (temporal) and *where* we look (spatial) sets the stage for the deeper dissociation explored later.

Local manifestations at the word level exhibit stage-dependent sensitivity. Early measures of lexical access (FFD, SFD, GD) exhibited lower sensitivity to the three-way interaction, indicating their automatic nature. Nevertheless, these early metrics were not immune to load. The analysis demonstrated that memory load significantly prolonged initial fixation durations, particularly for L2, aligning with the theory of limited cognitive resources [58], which posits that high load depletes attentional resources during initial encoding. Furthermore, the consistent L2 > L1 effect across all loads in these early measures confirms the theory of non-equilibrium bilingual representation [59], highlighting the inherent resource demands of L2 lexical access even at the initial stage. This establishes a baseline of general load and language effects against the more specific, interactive effects in later processing stages.

The three-way interaction significantly influenced late-stage processes involving semantic integration, conflict resolution, and reanalysis. Metrics such as RPD, FD-TW, RC, SC-TW, and FC-TW collectively demonstrate a processing sequence of “memory load → resource allocation → strategy adjustment”. High memory load depletes capacity, leading to longer regression and fixation durations, which impair semantic integration and force representational reconstruction [26]. Critically, the language‑context interaction observed across these metrics revealed a pattern consistent with the resource‑adaptive framework rather than the production‑based inhibitory control model. For L1, switch effects were consistently negative across these late‑stage metrics (*ps* < 0.001 vs. 0), indicating switch benefits (i.e., fewer regressions and fixations in switch trials). For L2, switch effects were consistently positive across these metrics (*ps* < 0.001 vs. 0), indicating switch costs (i.e., more regressions and fixations in switch trials). This asymmetry, with L1 benefiting and L2 incurring costs during semantic reanalysis, suggests that the dominant language, once accessed, can be integrated more efficiently, whereas the nondominant language demands additional processing resources even at late stages of comprehension. Moreover, load modulated these effects in a manner consistent with the Adaptive Control Hypothesis [11]: under high load, the L2 cost was most pronounced, reflecting the depletion of resources needed for controlled semantic integration; the L1 benefit remained robust, indicating that automated processing is more resilient to resource constraints. The observed pattern indicates that resource constraints primarily affect controlled, proactive mechanisms (such as planning saccades and maintaining semantic predictions), which in turn lead to subsequent corrective adjustments in representation via fixations and regressions. This highlights the hierarchical and resource‑sensitive nature of late‑stage processing under cognitive load.

The physiological metric for late-stage processing provides further insights that converge with this resource-adaptive interpretation. Blink count on the target word (BC-TW), an index of attentional engagement [25], displayed high sensitivity to context and language-context interactions. The observed pattern showed that for L1, blinks were less frequent in switch contexts than in non-switch contexts, whereas for L2, blinks were more frequent in switch contexts. This pattern aligns with the cost-benefit asymmetry observed in the late-stage eye-movement metrics: for L1, switching into the dominant language conferred a processing benefit, accompanied by reduced blinking (i.e., more stable attentional engagement); for L2, switching into the nondominant language incurred a processing cost, accompanied by increased blinking (i.e., heightened attentional demands). The increased blinking for L2 during switches highlights the substantial attentional resources required for semantic integration of the nondominant language under switching demands, consistent with the resource-adaptive framework [11]. Together, these physiological data corroborate that the late-stage processing asymmetry, benefits for L1 and costs for L2, is not merely a reflection of response strategies but is grounded in differential attentional and cognitive effort.

### Asymmetric and adaptive modulation of language switch cost

The behavioral and oculomotor data converge on a core principle: the cognitive cost of switching languages is not fixed but is asymmetrically and adaptively modulated by available working memory resources. Importantly, this pattern must be interpreted within the production-comprehension distinction. Classic switch cost asymmetries, larger costs for the dominant language, have been consistently observed in production tasks, where the Inhibitory Control Model [4] explains them as a result of actively inhibiting the dominant language [7, 8]. In contrast, comprehension studies often report more variable patterns, including reduced or symmetrical costs, and even switch benefits [9, 10]. Our findings align with the comprehension-specific literature, extending it by showing that the direction and magnitude of switch effects are further modulated by available working memory resources.

In the current study, a striking asymmetry emerged: under high load, switching into L1 resulted in a significant switch cost, whereas switching into L2 consistently yielded switch benefits across all load levels. This pattern suggests that the mechanisms underlying language switching in comprehension differ qualitatively from those in production. In comprehension, the language of the incoming word itself serves as a salient contextual cue. When cognitive resources are abundant, the system may flexibly process both languages. However, under high load, switching into the dominant L1 may be costly because it requires disengaging from the L2 that was just processed, a process that demands resources. Conversely, switching into the non-dominant L2 may be beneficial because it reduces competition from the always-active L1, effectively serving as a form of “cognitive unloading” [13]. This interpretation is consistent with the Adaptive Control Hypothesis [11], which emphasizes the context-dependent and resource-adaptive nature of bilingual language control.

Converging physiological evidence from pupil size and blink count per sentence further supports for this interpretation. Pupil dilation, a well-established indicator of cognitive effort [21], indicated that both the L1 non-switch context and the L2 switch context elicited the largest pupil responses under high load. This aligns with the behavioral asymmetry: switching into L1 under high load required effortful disengagement from the active L2, whereas switching into L2 under high load imposed a processing burden despite the observed behavioral benefit. The blink rate, which reflects attentional engagement [25], showed a complementary pattern: blinks were more frequent for L1 in non-switch contexts (reflecting sustained monitoring of the dominant language) and for L2 in switch contexts (reflecting heightened attention during language switching). Together, these physiological measures corroborate that the asymmetric switch effects observed in reaction times and eye movements are accompanied by differential autonomic and attentional demands, consistent with the resource-adaptive framework.

The differential regulation mode is further supported by the distinct cognitive characteristics of L1 and L2 processing. L1 processing is predominantly automated, requiring fewer executive control resources [60]. When the working memory system is engaged in high-load tasks, the resources available for inhibiting native language activation become limited, leading to greater disruption in L1-switching contexts. L2 processing, by contrast, relies more heavily on controlled, resource-demanding processes [15]. Under high load, bilingual individuals may engage in a “full control” processing mode, where switching into L2 becomes a streamlined, cue-driven operation that reduces task difficulty. This adaptive behavior implies that the “cost” of language switching is not a fixed property but a consequence of cognitive system adjustments under specific resource constraints, indicating that language switching can serve as a processing cue in particular research contexts [3].

### Temporal-spatial dissociation in eye movements and theoretical implications

Beyond quantifying interaction effects, our eye-tracking data revealed a fundamental dissociation between the temporal and spatial dimensions of oculomotor control during loaded bilingual reading. This dissociation provides unique, process-pure insights into the architecture of bilingual language control.

Across all load levels, the temporal dimension, indexed by FDpS, consistently showed that switching into L1 conferred a processing benefit (shorter fixations in switch trials), whereas switching into L2 incurred a processing cost (longer fixations in switch trials). In stark contrast, the spatial dimension, indexed by SCpS and FCpS, exhibited the opposite pattern: switching into L1 incurred a cost (more saccades and fixations in switch trials), whereas switching into L2 conferred a benefit (fewer saccades and fixations in switch trials). This cross-dimensional asymmetry suggests that the cognitive system treats the two languages fundamentally differently depending on whether one measures when the eyes move (temporal efficiency) or where they move (spatial allocation).

Notably, both dimensions showed convergent load modulations at the medium load level. For L1, the temporal benefit and the spatial cost were both largest under medium load. For L2, the temporal cost and the spatial benefit were also largest under medium load. This convergence at medium load, where the L1 benefit, L1 cost, L2 cost, and L2 benefit all reached their maxima, suggests that medium load represents a critical window of maximum competition between language control and semantic integration [1]. Under this condition, the system must allocate resources simultaneously to inhibit the dominant language (for L2 switches) and to resolve conflict from the non-dominant language (for L1 switches), resulting in heightened activity across both temporal and spatial domains.

Under high load, by contrast, the spatial benefit for L2 diminished significantly (e.g., SCpS: *p* < .01, FCpS: *p* < .05), whereas the temporal cost for L2 showed a non-significant reduction. This pattern suggests that the two dimensions exhibit differential vulnerability to resource depletion. The spatial dimension, which relies more heavily on proactive planning (e.g., parafoveal preview and saccadic targeting), appears particularly sensitive to high cognitive load, as reflected in the significant decline of the L2 spatial benefit. In contrast, the temporal dimension, which arguably reflects more automatic and online processing, proved more robust, with the L2 temporal cost remaining relatively stable even under high load. Thus, rather than a strategic trade-off between dimensions, the data point to an asymmetric susceptibility of the underlying oculomotor subsystems to cognitive resource constraints.

Early spatial processing strategies, indexed by word skipping rate (SR), further illuminated the baseline dissociation between L1 and L2. The significantly higher skipping rate observed for L1 aligns with the lexical access threshold theory [61], indicating that the more automated L1 representations can often be identified via parafoveal preview, eliminating the requirement for direct fixation. Conversely, the lower skipping rate in L2 underscores its dependence on foveal processing. Furthermore, the reduction in skipping rate during switch contexts, regardless of the specific language, supports the language monitoring hypothesis [62], suggesting that the switching task itself depletes the preview resources necessary for efficient skipping, as attention is allocated to monitoring the language cue.

A systematic comparison of eye-tracking measures demonstrates a consistent dissociation in how switching affects the two languages. In the temporal domain, L2 consistently exhibits switch costs, indicated by longer processing times in switch trials across early (SFD, GD), late (RPD, FD-TW), and physiological (BCpS, BC-TW) measures, as well as in the global reading measure FDpS. Conversely, L1 shows switch benefits in FDpS and in late-stage measures (RPD, FD-TW, RC, SC-TW, FC-TW), suggesting that switching into the dominant language enhances semantic integration. In the spatial domain, L1 consistently demonstrates switch costs with more saccades and fixations in SCpS, FCpS, and pupil size (PS), while L2 shows switch benefits in SCpS and FCpS. Two measures, rightward saccade distance (RSD) and skipping rate (SR), show no reliable switch effects for either language. The ANOVA for RSD reveals no significant main effects or interactions, and similarly, no significant effects are found for SR. This pattern across measures highlights a fundamental asymmetry: the non-dominant language incurs temporal and attentional costs across most processing stages, while the dominant language benefits temporally but experiences spatial costs. This dissociation aligns with the resource-adaptive framework and underscores the distinct roles of automaticity and controlled processing in bilingual comprehension.

Taken together, these findings challenge the notion of bilingual language control as a singular, domain-general function. Instead, they support multi-component or hierarchical models in which distinct cognitive sub-processes—such as a “temporal integration loop” for meaning assembly and a “spatial attention controller” for visual sampling [18, 63]—can be selectively engaged and differentially affected by cognitive load [64]. Our data indicate that memory load and language switching disrupt the coordination between these subsystems. Switch effects reveal at least two separable sub-costs: a temporal integration cost, observed as prolonged fixations and regressions, and a spatial attentional cost, characterized by disorganized scanpaths. Language dominance and late-stage semantic integration significantly influence the temporal integration cost, whereas contextual predictability and cognitive load more strongly impact the spatial attentional cost.

This dissociation provides a valuable target for future neuroimaging studies in cognitive neuroscience. Researchers are encouraged to investigate distinct neural correlates, such as exploring potential correlations between temporal integration costs (e.g., RPD, FD-TW) and activation in left-lateralized language networks (e.g., the left inferior frontal gyrus) [65] and default-mode network regions associated with semantic memory [66]. Similarly, spatial attentional costs (e.g., SCpS, saccade amplitude) could be linked to activity in the dorsal attention network (e.g., the frontal eye fields and the intraparietal sulcus) [67]. The integration of eye-tracking with fMRI or EEG methodologies offers a promising approach to directly map the dual temporal-spatial processing onto specific brain networks [68].

This study bridges the gap between language-specific switching processing and domain-general cognitive control research by introducing the variable of memory load. Previous studies have shown the impact of working memory load on neural activity in various domains, including executive function and task-switching (e.g., n-back paradigms) [69], conflict monitoring and inhibitory control (e.g., Stroop and Flanker tasks) [17], and visuospatial and verbal working memory [16]. Our research expands this framework to language switching, revealing a significant interplay between language control mechanisms and the working memory system. This advancement shifts the focus from the debate on “whether there is a switching cost” to a more profound investigation of “when, under what conditions, and in what form the switching cost manifests” [18].

### Limitations and future research directions

Despite the valuable findings obtained from this study, there are some limitations as follows.

Specifically, the homogeneity of the participant group, consisting of bilingual individuals with advanced educational backgrounds in both Chinese and English, may impact the generalizability of the results. Previous research suggests that the effects of memory load modulation may vary among bilinguals with differing proficiency levels [18], age groups, or language-switching habits [70, 71]. Future studies could consider recruiting a more diverse range of participants to enhance the applicability of the conclusions.

The use of a visual memory recognition task in this study to evaluate working memory load underscores the importance of considering different types of loads, such as spatial load, which may have varying effects on language processing [72]. Future research could systematically compare the modulatory effects of different loads further to explore the relationship between cognitive resources and language processing.

Additionally, limitations in the research methodology include the inability of eye-tracking technology to directly monitor neural activity. Future studies could integrate eye-tracking with electroencephalography (EEG) or functional near-infrared spectroscopy (fNIRS) to improve comprehension [73–75]. Examining neural oscillations in the theta and alpha bands, which are associated with working memory and attention control [76, 77], may offer deeper insights into the neurocognitive mechanisms underlying memory load in language code switching.

## Conclusion

This study employed eye-tracking technology to investigate the intricate regulatory mechanisms underlying language switching during sentence comprehension under working memory load. The research identified a dynamic and multidimensional regulatory effect influenced by language direction, processing stage, and cognitive resource allocation. The key finding emphasized the consistent regulatory impact of working memory load on bilingual reading across various levels and processing stages, supporting the Adaptive Control Hypothesis [11]. Specifically, the study demonstrated that native-language processing relies on automated processes, necessitating basic conflict monitoring even in non-switching contexts, whereas L2 processing is influenced by dynamic resource allocation, resulting in a pattern of temporal costs but spatial benefits in switching contexts. Discrepancies in performance between L1 and L2 under varying loads reveal the cognitive system’s dynamic balance in optimizing L1 monitoring and compensating for L2 inhibition, elucidating the cognitive dissociation observed in bilingual switching during sentence comprehension.

## Supplementary Information

Supplementary Material 1

## Data Availability

Data and materials can be obtained upon reasonable request from the first author.

## Abbreviations

T1: Task 1 (sentence judgment)
T2: Task 2 (string recognition)
FDpS: Fixation duration per sentence
RSD: Rightward saccade distance
SCpS: Saccade count per sentence
FCpS: Fixation count per sentence
PS: Pupil size
BCpS: Blink count per sentence
FFD: First fixation duration
SFD: Single fixation duration
GD: Gaze duration
SR: Skipping rate
RPD: Regression path duration
FD-TW: Fixation duration on the target word
RC: Regression count
SC-TW: Saccade count on the target word
FC-TW: Fixation count on the target word
BC-TW: Blink count on the target word
RT: Reaction time
LL: Low load
ML: Medium load
HL: High load
C-NSC: Chinese non-switch context
C-SC: Chinese switch context
E-NSC: English non-switch context
E-SC: English switch context
