# Supplementary material for: The modulating role of memory load on language switching in sentence comprehension: evidence from eye movements
Source: BMC Psychol. 2026 May 30;14:880. doi: 10.1186/s40359-026-04871-1 (PMC13274029; doi:10.1186/s40359-026-04871-1)
Supplement: Supplementary file 1 — Supplementary Material 1 [file 40359_2026_4871_MOESM1_ESM.docx]

**Supplementary materials: Tables S1—S6 and Figs. S1—S6**

**Table S1** RSD (amplitudes) and the switch effects under various conditions

|  | C-NSC | C-SC | L1 effects | E-NSC | E-SC | L2 effects |
| --- | --- | --- | --- | --- | --- | --- |
| LL | 7.06±1.85 | 6.80±2.06 | 0.26±1.91 | 6.49±1.96 | 5.94±1.78 | 0.55±1.72 |
| ML | 7.29±1.93 | 6.97±1.82 | 0.32±1.60 | 6.89±2.27 | 5.96±1.77 | 0.93±2.33 |
| HL | 7.44±1.84 | 7.10±1.86 | 0.34±1.69 | 6.79±2.05 | 5.90±1.75 | 0.89±2.18 |

Switch effects for RSD were calculated as non-switch minus switch, following the unified principle described in the Data Analysis section

**Table S2** FCpS (times) and the switch effects under various conditions

|  | C-NSC | C-SC | L1 effects | E-NSC | E-SC | L2 effects |
| --- | --- | --- | --- | --- | --- | --- |
| LL | 11.38±1.58 | 12.52±1.67 | 1.14±1.00 | 13.03±1.46 | 11.69±1.51 | -1.34±1.06 |
| ML | 11.30±1.60 | 12.49±1.71 | 1.19±1.05 | 12.89±1.62 | 11.38±1.53 | -1.51±0.98 |
| HL | 11.52±1.66 | 12.37±1.81 | 0.85±0.98 | 12.73±1.56 | 11.50±1.68 | -1.23±1.00 |

FCpS effects = FCpS in switch context – FCpS in non-switch context

**Table S3** BCpS (times) and the switch effects under various conditions

|  | C-NSC | C-SC | L1 effects | E-NSC | E-SC | L2 effects |
| --- | --- | --- | --- | --- | --- | --- |
| LL | 2.26±0.95 | 2.17±0.95 | -0.09±0.37 | 2.04±0.84 | 2.24±0.96 | 0.20±0.43 |
| ML | 2.18±0.91 | 2.10±0.91 | -0.08±0.26 | 2.02±0.85 | 2.16±0.93 | 0.14±0.33 |
| HL | 2.13±0.94 | 2.00±0.90 | -0.13±0.29 | 1.98±0.95 | 2.08±0.99 | 0.10±0.25 |

BCpS effects = BCpS in switch context – BCpS in non-switch context

**Table S4** SFD (ms) and the switch effects under various conditions

|  | C-NSC | C-SC | L1 effects | E-NSC | E-SC | L2 effects |
| --- | --- | --- | --- | --- | --- | --- |
| LL | 278.79±146.25 | 226.12±87.95 | -52.67±173.69 | 253.19±70.22 | 272.87±100.33 | 19.68±121.78 |
| ML | 286.76±130.92 | 217.41±52.60 | -69.35±131.01 | 263.32±63.53 | 338.82±156.66 | 75.50±159.91 |
| HL | 284.78±109.48 | 249.44±101.47 | -35.34±148.21 | 250.62±58.40 | 362.27±150.94 | 111.65±150.12 |

SFD effects = SFD in switch context – SFD in non-switch context

**Table S5** RC (times) and the switch effects under various conditions

|  | C-NSC | C-SC | L1 effects | E-NSC | E-SC | L2 effects |
| --- | --- | --- | --- | --- | --- | --- |
| LL | 1.59±0.26 | 1.27±0.18 | -0.32±0.32 | 1.23±0.20 | 1.60±0.32 | 0.37±0.29 |
| ML | 1.66±0.48 | 1.19±0.16 | -0.47±0.46 | 1.24±0.18 | 1.66±0.32 | 0.42±0.32 |
| HL | 1.66±0.39 | 1.22±0.21 | -0.44±0.39 | 1.22±0.16 | 1.65±0.34 | 0.43±0.33 |

RC effects = RC in switch context – RC in non-switch context

**Table S6** SC-TW (times) and the switch effects under various conditions

|  | C-NSC | C-SC | L1 effects | E-NSC | E-SC | L2 effects |
| --- | --- | --- | --- | --- | --- | --- |
| LL | 5.83±1.04 | 4.62±0.99 | -1.21±1.14 | 4.27±0.85 | 6.76±1.12 | 2.49±1.05 |
| ML | 6.04±0.95 | 4.58±1.09 | -1.46±1.12 | 4.42±1.00 | 7.02±1.12 | 2.60±1.06 |
| HL | 6.12±1.04 | 4.63±1.32 | -1.49±1.21 | 4.36±0.79 | 7.27±1.11 | 2.91±1.06 |

SC-TW effects = SC-TW in switch context – SC-TW in non-switch context


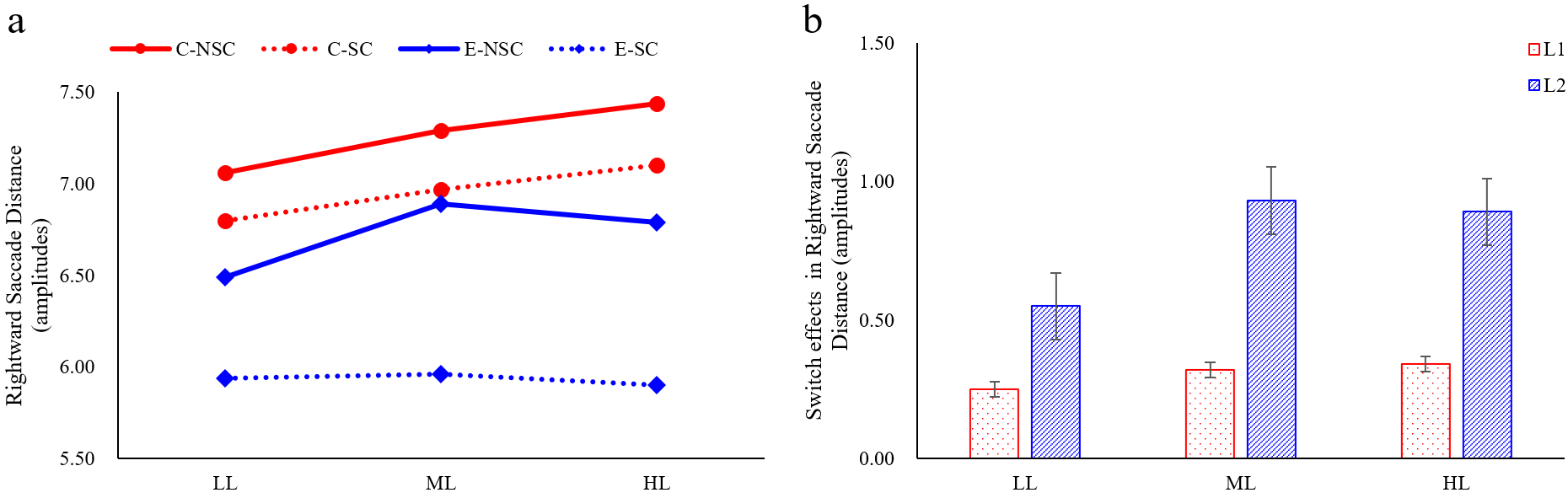


Fig. S1 The distribution patterns of RSD (a) and its switch effects (b)


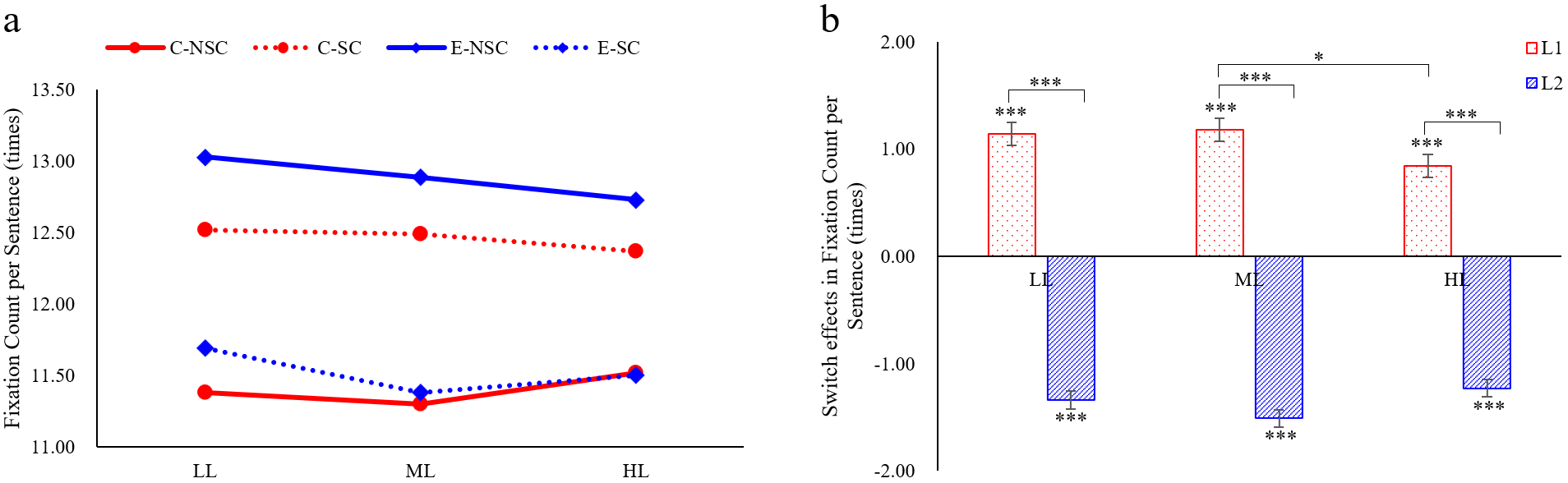


Fig. S2 The distribution patterns of FCpS (a) and its switch effects (b)


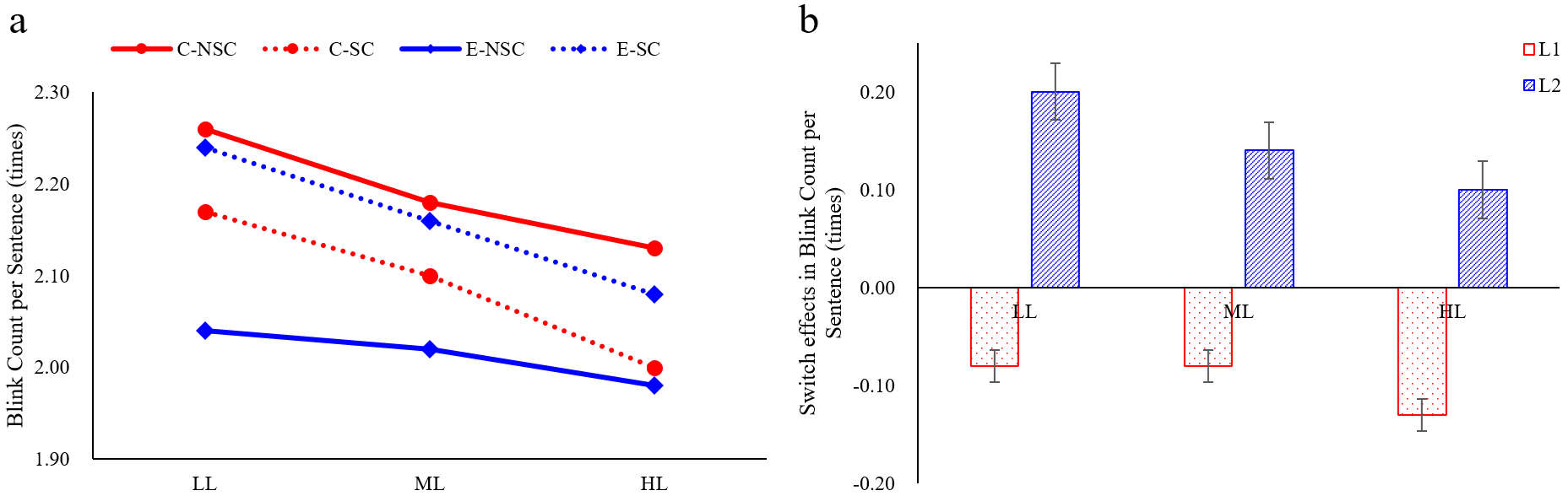


Fig. S3 The distribution patterns of BCpS (a) and its switch effects (b)


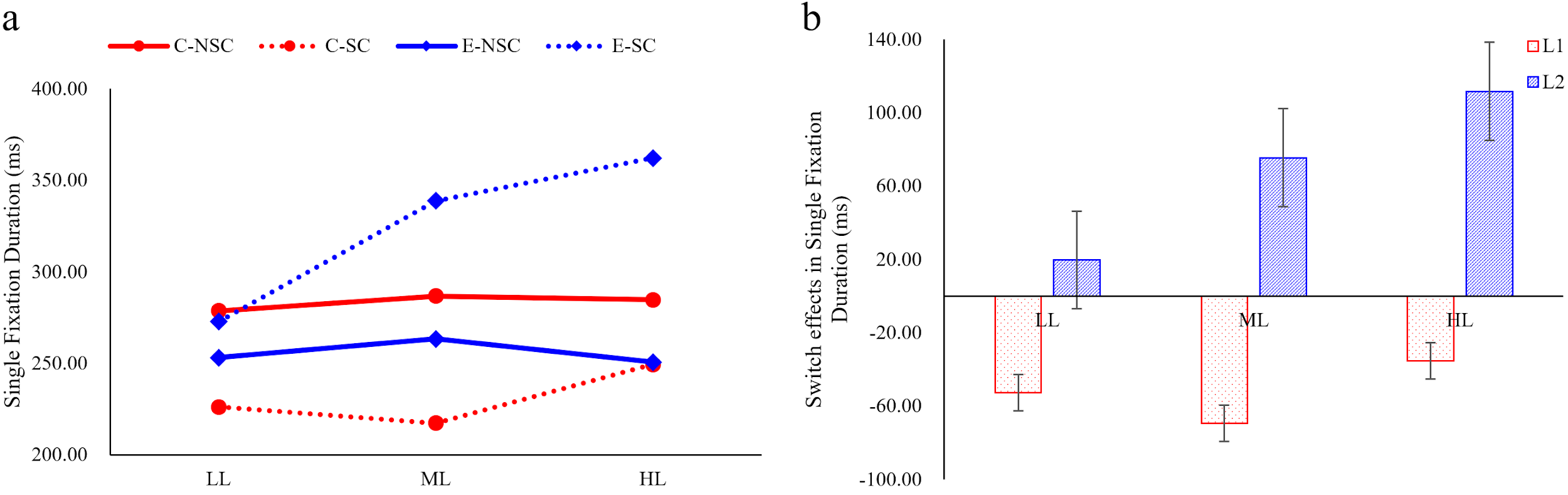


Fig. S4 The distribution patterns of SFD (a) and its switch effects (b)


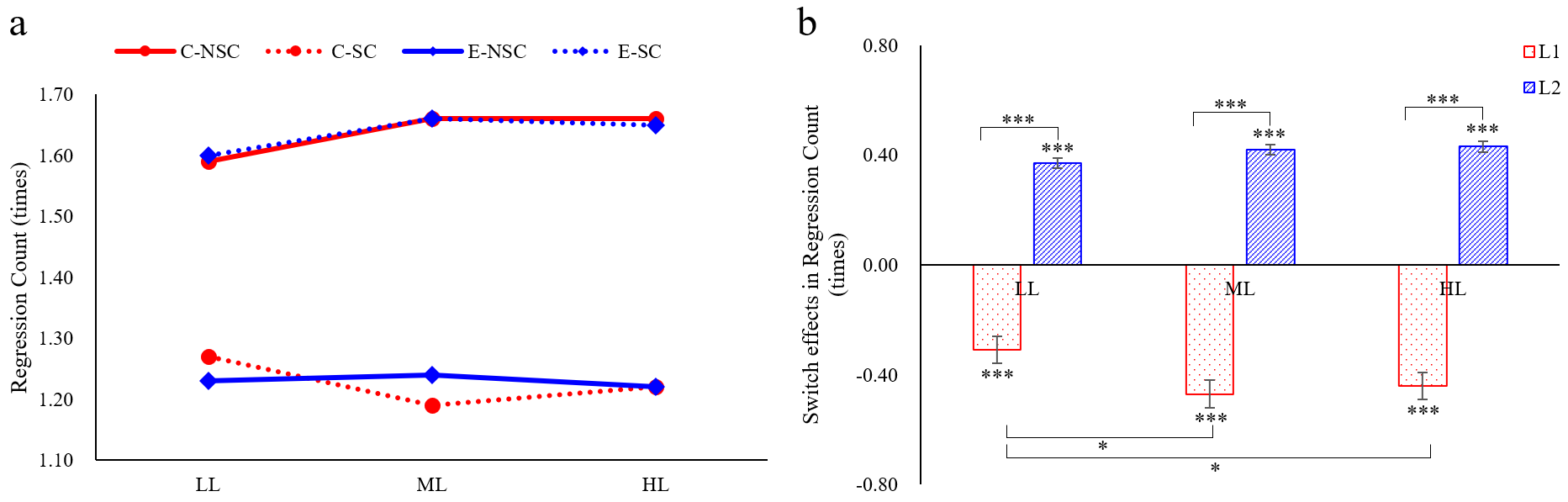


Fig. S5 The distribution patterns of RC (a) and its switch effects (b)


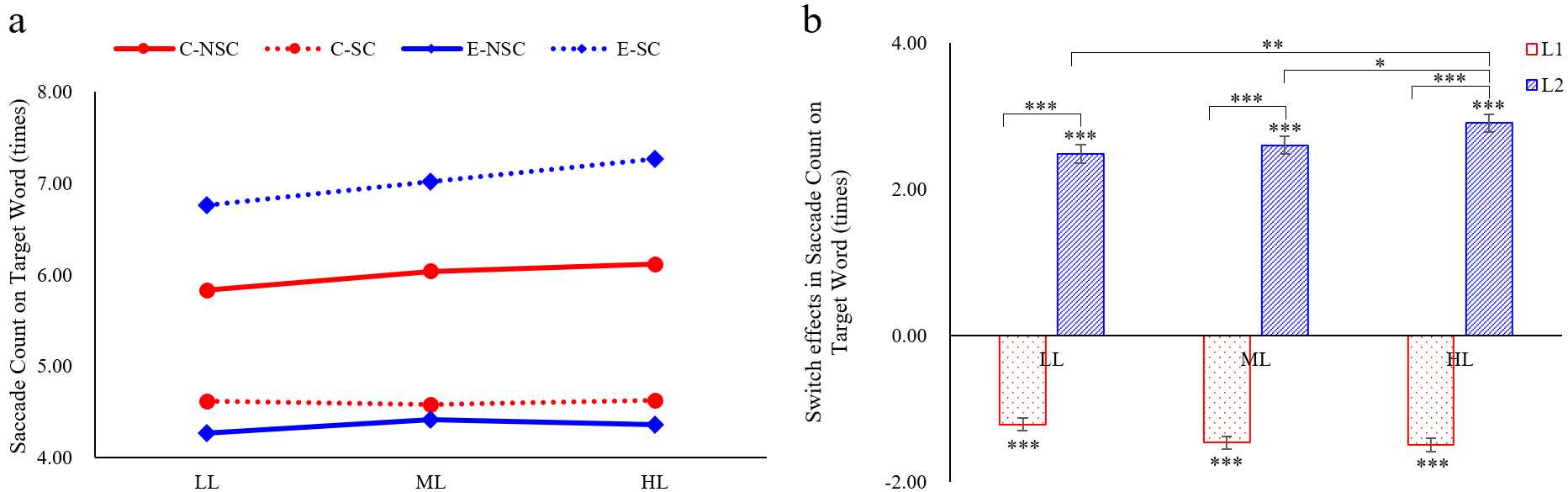


Fig. S6 The distribution patterns of SC-TW (a) and its switch effects (b)
